# Genomic Insights into the Origin of Parasitism in the Emerging Plant Pathogen *Bursaphelenchus xylophilus*

**DOI:** 10.1371/journal.ppat.1002219

**Published:** 2011-09-01

**Authors:** Taisei Kikuchi, James A. Cotton, Jonathan J. Dalzell, Koichi Hasegawa, Natsumi Kanzaki, Paul McVeigh, Takuma Takanashi, Isheng J. Tsai, Samuel A. Assefa, Peter J. A. Cock, Thomas Dan Otto, Martin Hunt, Adam J. Reid, Alejandro Sanchez-Flores, Kazuko Tsuchihara, Toshiro Yokoi, Mattias C. Larsson, Johji Miwa, Aaron G. Maule, Norio Sahashi, John T. Jones, Matthew Berriman

**Affiliations:** 1 Forestry and Forest Products Research Institute, Tsukuba, Japan; 2 Parasite Genomics, Wellcome Trust Sanger Institute, Wellcome Trust Genome Campus, Hinxton, United Kingdom; 3 Molecular Biosciences-Parasitology, Institute of Agri-Food and Land Use, School of Biological Sciences, Medical Biology Centre, Queen's University Belfast, Belfast, United Kingdom; 4 College of Bioscience and Biotechnology, Chubu University, Kasugai, Japan; 5 Neurosensing and Bionavigation Research Center, Doshisha University, Kyotanabe, Kyoto, Japan; 6 Plant Pathology Programme, The James Hutton Institute, Invergowrie, United Kingdom; 7 Department of Plant Protection Biology, Division of Chemical Ecology, Swedish University of Agricultural Sciences, Alnarp, Sweden; Virginia Polytechnic Institute and State University, United States of America

## Abstract

*Bursaphelenchus xylophilus* is the nematode responsible for a devastating epidemic of pine wilt disease in Asia and Europe, and represents a recent, independent origin of plant parasitism in nematodes, ecologically and taxonomically distinct from other nematodes for which genomic data is available. As well as being an important pathogen, the *B. xylophilus* genome thus provides a unique opportunity to study the evolution and mechanism of plant parasitism. Here, we present a high-quality draft genome sequence from an inbred line of *B. xylophilus*, and use this to investigate the biological basis of its complex ecology which combines fungal feeding, plant parasitic and insect-associated stages. We focus particularly on putative parasitism genes as well as those linked to other key biological processes and demonstrate that *B. xylophilus* is well endowed with RNA interference effectors, peptidergic neurotransmitters (including the first description of *ins* genes in a parasite) stress response and developmental genes and has a contracted set of chemosensory receptors. *B. xylophilus* has the largest number of digestive proteases known for any nematode and displays expanded families of lysosome pathway genes, ABC transporters and cytochrome P450 pathway genes. This expansion in digestive and detoxification proteins may reflect the unusual diversity in foods it exploits and environments it encounters during its life cycle. In addition, *B. xylophilus* possesses a unique complement of plant cell wall modifying proteins acquired by horizontal gene transfer, underscoring the impact of this process on the evolution of plant parasitism by nematodes. Together with the lack of proteins homologous to effectors from other plant parasitic nematodes, this confirms the distinctive molecular basis of plant parasitism in the *Bursaphelenchus* lineage. The genome sequence of *B. xylophilus* adds to the diversity of genomic data for nematodes, and will be an important resource in understanding the biology of this unusual parasite.

## Author Summary

*Bursaphelenchus xylophilus* is an important plant pathogen, responsible for an epidemic of pine wilt disease in Asia and Europe. *B. xylophilus* has acquired the ability to parasitise plants independently from other economically important nematodes and has a complex life cycle that includes fungal feeding and a stage associated with an insect, as well as plant parasitism. We have sequenced the genome of *B. xylophilus* and used it as a resource to understand disease mechanisms and the biological basis of its complex ecology. The ability to break down cellulose, the major component of the plant cell wall, is a major problem for plant parasitic nematodes as few animals can produce the required enzymes (cellulases). Previous work has shown that other plant parasitic nematodes have acquired cellulases from bacteria but we show that all *Bursaphelenchus* cellulases were most likely acquired independently from fungi. We also describe a complex set of genes encoding enzymes that can break down proteins and other molecules, perhaps reflecting the range of organisms with which *B. xylophilus* interacts during its life cycle. The genome sequence of *Bursaphelenchus* represents an important step forward in understanding its biology, and will contribute to efforts to control the devastating disease it causes.

## Introduction

The nematode *Caenorhabditis elegans* was the first multicellular organism for which a complete genome sequence was available, and subsequent genomics research on a wider range of nematodes has provided information on many important biological processes and is underpinned by the information developed for *C. elegans* [1]. While *C. elegans* is a free-living bacterial feeder, nematodes exhibit a wide range of ecological interactions, including important parasites of humans and livestock that have huge agricultural and medical impacts [2].

Plant parasitic nematodes cause damage to crops globally. Within the Nematoda, the ability to parasitise plants has evolved independently on several occasions [3] and nematodes use a wide range of strategies to parasitise plants. Some nematodes are migratory ectoparasites which remain outside the roots and have a very limited interaction with their hosts. Migratory endoparasitic nematodes invade their host and cause extensive damage as they move through the host and feed. Sedentary endoparasitic nematodes, including the cyst nematodes and root knot nematodes, have highly complex biotrophic interactions with their hosts. These are the most damaging plant nematodes and consequently genomes for two species of root knot nematode have been reported [4], [5] with others in progress for cyst nematodes. Currently there is no genome sequence for any migratory endoparasitic nematode.

The pine wood nematode *Bursaphelenchus xylophilus* is a migratory endoparasite that causes severe damage to forestry and forest ecosystems (reviewed in [6]). *B. xylophilus* is native to North America such that trees there have evolved tolerance or resistance to the pathogen. However, at the start of the 20^th^ Century it was introduced into Japan and has subsequently spread to other countries in Asia where no natural resistance is present, causing huge damage in an on-going epidemic of pine wilt disease. Despite global quarantine efforts *B. xylophilus* was recently introduced into Portugal [7] and has now also spread to Spain.

**Figure 1:**
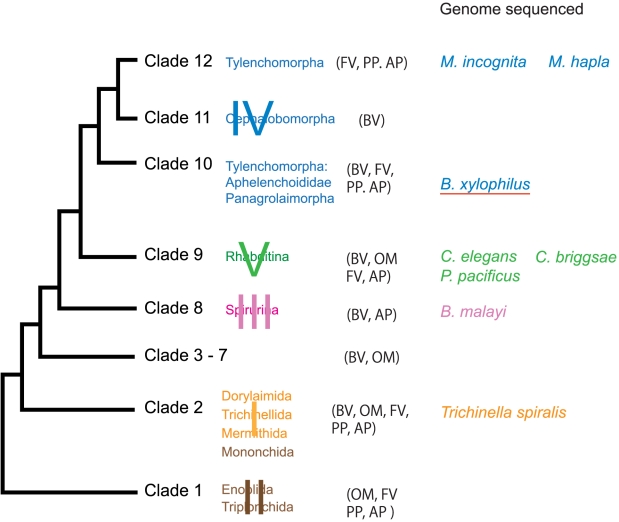
Major taxonomic groups of the phylum Nematoda and species whose genomes sequenced. The major clades of Blaxter et al [89] are labeled I, II, III, IV, V and minor clades of van Megen [3] have Arabic numeral identifiers (1 to 12). The trophic mode indicated by the following abbreviations [90]: AP, animal parasite; BV, bacteriovore; FV, fungivore; OM, omnivore; PP, plant parasite.

**Figure 2:**
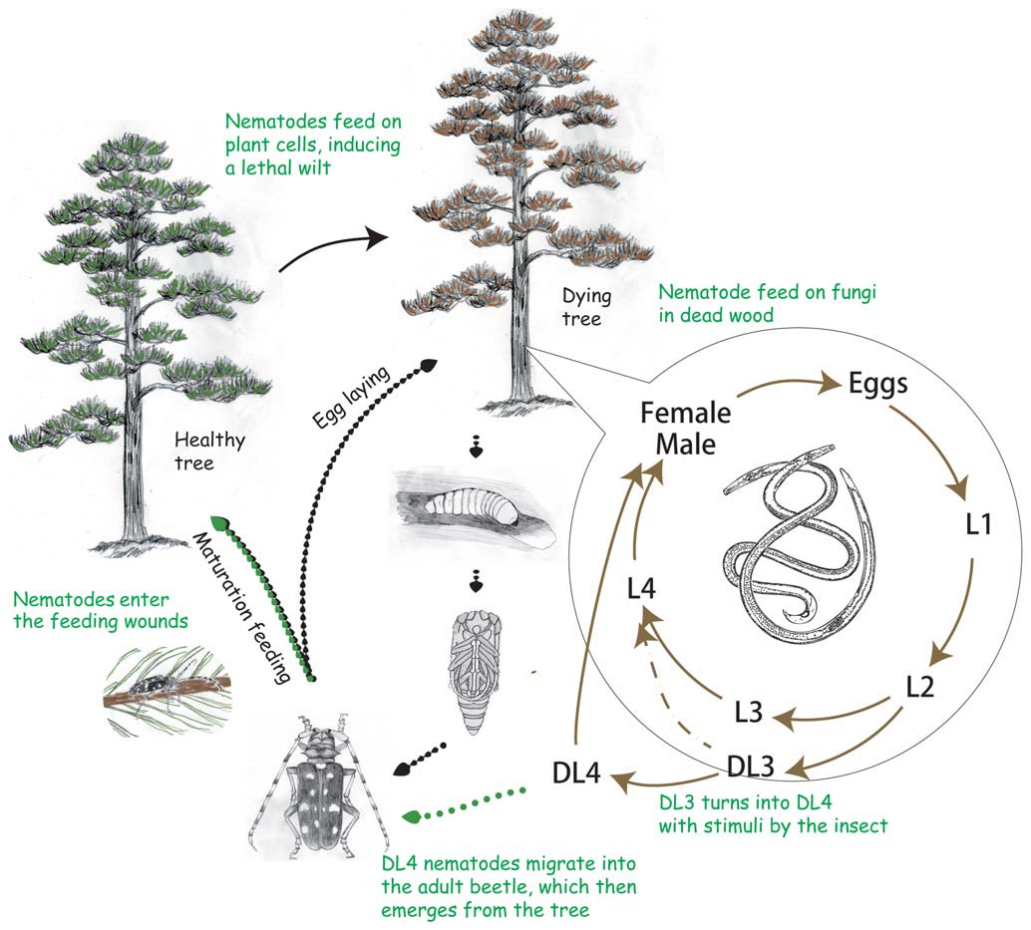
The life cycle of the pine wood nematode *Bursaphelenchus xylophilus*. The nematode developmental cycles are shown with brown arrows. The nematodes develop through four moults (ie. four larval stages L1, L2, L3, L4 and adult) and reproduce within wood tissue while food is available. When conditions are adverse (ie. food becomes limiting) *B. xylophilus* enters specialized third-stage dauer larva (DL3). When stimulated by the presence of the vector beetle, the DL3 molts to become the fourth-stage dispersal larva (DL4) in preparation to board the vector. As the adult beetle emerges the nematodes move and settle beneath the elytra or within the trachea of the beetles and are transported to another food source. After invading healthy trees the nematodes feed on parenchymal cells and migrate through the tissues to spread over the tree, leading to wilting symptoms that result in the death of the tree within a year of infection. When the tree is dying *B. xylophilus* feeds on fungi which invade the tree and reproduces quickly.

Most species within the *Bursaphelenchus* genus, including the closest relatives of *B. xylophilus* [8], are fungal feeders that are transmitted by vector insects only to dead or dying trees during oviposition. *B. xylophilus* and the few other pathogenic species described to date are unique in their capacity to feed on live trees as well as on the fungi that colonise dead or dying trees, so these species may represent a relatively recent, independent origin of plant parasitism. The nematode is a member of the Aphelenchoididae and belongs to clade 10 [3] while most other major plant parasites including *Meloidogyne* species belong to clade12 [3] (Figure 1). The life cycle of *B. xylophilus* is summarised in Figure 2 and the infection and disease process has been reviewed by Mamiya [9] and by Jones *et al.* [6].

Little was known about the molecular basis of the interactions between *B. xylophilus* and its host plants. A series of advances have been made in the last few years, underpinned by a relatively small scale Expressed Sequence Tag (EST) project on this nematode [10]. Genes involved in parasitism were identified and characterised, including those encoding a wide range of plant cell wall degrading or modifying proteins [11]–[13]. As in other plant parasitic nematodes, it is now clear that horizontal gene transfer has played an important role in the evolution of plant parasitism in *B. xylophilus* [14].

In order to shed further light on the mechanisms of parasitism used by *B. xylophilus* and to investigate the genetic and genomic factors involved in the evolution of parasitism, we have produced a high quality genome sequence from an inbred line of this nematode. We describe the assembly and initial annotation and characterisiation of the genome sequence, then interrogate this dataset to identify genes involved in key biological processes, including those associated with chemosensation, neurotransmission, alimentation, stress-responses and development. Further, we identify genes potentially important in functional genetics such as the RNAi pathway and putative control targets such as G-protein coupled receptors (GPCRs), peptidases and neuropeptide genes. This genome sequence allows a comparison of genes involved in plant parasitism across nematode clades and expands our knowledge of the role played by horizontal gene transfer in the evolution of plant parasitism by nematodes.

## Materials and Methods

### Biological materials and DNA/RNA extraction

The *B. xylophilus* Ka4 population, which originated in Ibaraki prefecture Japan and has been maintained for over 15 years in the Nematology Lab in FFPRI was previously used to generate biological material for ESTs. The Ka4C1 inbred line was established by sister-brother mating over 8 generations from the Ka4 population and was used to generate of material for genome sequencing.

Nematodes were cultivated for 10 days on *Botrytis cinerea* grown on autoclaved barley grains with antibiotics (100 µg/ml streptomycin and 25 µg/ml chloramphenicol). The nematodes were collected using a modified Baermann funnel technique for 3 h at 25°C and cleaned by sucrose flotation [15] followed by three rinses in 1x PBST. The cleaned nematodes were incubated in 1x PBST containing antibiotics (100 µg/ml streptomycin and 25 µg/ml chloramphenicol) at 23°C for 8 hours to allow nematodes to digest fungal residues remaining in their guts before use. Genomic DNA was extracted from nematodes using GenomeTip-100G (Qiagen) following the manufacturer's instructions.

Poly-(A) + RNA was extracted from mixed-stage nematodes or fourth-stage dispersal larva (DL4 or LIV) collected from the vector insect beetle as described previously [10] and used for the construction of EST libraries.

### Chromosome number and genome size estimation

To determine the *B. xylophilus* chromosome number, chromosomes were observed in early embryos by DAPI staining and confocal laser-scanning microscopy [16]. The genome size of *B. xylophilus* was estimated using real time PCR as described in Welhen et al [17]. Translation elongation factor 1 alpha (genbank no GU130155) was used as a reference. DNA concentration was calculated using Qubit (Invitrogen) and the real time PCR reaction was performed using the StepOnePlus system (Applied BioSystems) with SYBR Green I. These protocols can be extremely sensitive and consequently the experiments were repeated in triplicate.

### Sequencing strategy

#### 454/Roche GS FLX (454 FLX) sequencing

A library of single stranded DNA fragments was obtained from *B. xylophilus* genomic DNA using Roche/454 standard procedures and a total of 993,947 single reads were obtained from this library using a 454 FLX Genome Sequencer. For paired end sequencing, libraries of 8 kb and 20 kb fragments were prepared using the manufacturer's standard protocol after shearing using a Hydroshear device. A total of 1,729,870 reads for the 8 k library and 223,236 reads for the 20 k library were obtained after standard Roche quality trimming. Of these, 89% and 88% were recognized as paired by the Newbler assembler (version 2.3). The 454 FLX sequence data provided 13× coverage of the genome sequence.

#### Illumina sequencing

Genomic DNA was sequenced on Illumina Genome Analyser I (GAI) using standard procedures. The sequences were 51 bases long. A total of 71,593,088 reads passed the default filter, giving a total coverage of about 48 genome equivalents.

### Assembly process

Sequence data from 454 FLX and Illumina GAI were assembled using the Newbler *de novo* assembler (version 2.3) and Velvet assembler (version 1.0.12) [18] respectively. The result from each assembly was combined using Minimus2 (sourceforge.net/projects/amos/) and contigs supported by both assemblies were used as fake unpaired capillary reads in the subsequent Newbler assembly. The resulting assembly was improved using three different methods: AbacasII [19] to merge small contigs; IMAGE [20] to iteratively map and assemble short reads to close gaps; and iCORN [21] to iteratively correct single base substitutions and small indels.

As an indirect measure of completeness of the assembly, a search for orthologues was performed by CEGMA (ver. 2.0) software using CEGs (core eukaryotic genes); a set of 248 extremely highly conserved genes thought to be present in almost all eukaryotes in a reduced number of paralogues [22].

EST clustering was performed using PartiGene (ver. 3.0), a software pipeline designed to analyze and organize EST data sets [23]. Briefly, EST sequences were clustered into groups (putative genes) on the basis of sequence similarity and then clusters were assembled to yield consensus sequences using Phrap (P. Green, unpublished). Mapping of individual ESTs or clustered ESTs to the genome assembly was performed using PASA software [24].

### Protein coding gene prediction

EST data generated in this study and previously obtained by capillary sequencing [10] were used to predict genes in the assembly. The total number of ESTs used was 82,100.

A reference dataset of 565 *B. xylophilus* protein encoding genes was manually curated from EST clusters and predictions of highly conserved genes using CEGMA [25]. 365 of these were used to train *ab-initio* gene predictors Augustus [26] and SNAP [27] and 200 were used to evaluate accuracy of the predictions. EVidenceModeler [24] was used to combine all predictions from gene predictors Augustus, SNAP and GeneMark.hmm [28], EST mapping data from PASA [24] and protein homology data against the Pfam database using GeneWise2 [29]. Gene prediction accuracy was computed at the level of nucleotides, exons and complete genes on 200 manually-curated gene models (Figure S1) as described previously [30].

### Functional annotation

Initial functional annotation was performed using InterProScan to search against the InterPro protein family database, which included PROSITE, PRINTS, Pfam, ProDom, SMART, TIGRFAMs, PIR SuperFamily and SUPERFAMILY [31]. The latest version Pfam search (ver. 24.0) [32], which uses HMMER3 and is more sensitive than the previous version packed in InterProScan, was also performed independently for *B. xylophilus* genome. Gene Ontology annotation was derived using Blast2GO software [33] based on the BLAST match against NCBI non-redundant (NR) proteins with an E-value cutoff of 1e-10 and InterProScan results.

Assignments to conserved positions in metabolic and regulatory pathways were performed using KOBAS software [34] based on the KEGG annotation resource [35]. KEGG genes and KO term annotations were assigned based on similarity searches with a 1e-5 E-value cutoff and a rank cutoff 5. Significantly enriched pathway terms between two organisms were identified by frequencies of terms with chi-square and FDR correlation tests.

To study the evolution of gene families across nematodes within the order Rhabditida, we used the predicted protein sets from all 9 genomes available in WormBase release WS221 (www.wormbase.org) – *C. elegans*, *C. brenneri*, *C. briggsae*, *C. japonicum*, *C. remaneri*, *Meloidogyne hapla*, *M. incognita*, *Pristionchus pacificus* and *Brugia malayi*, together with predicted proteins of *B. xylophilus*. Version 2.0 of the OrthoMCL pipeline [36] was used to cluster proteins into families of orthologous genes, with default settings and the BLAST parameters recommended in the OrthoMCL documentation. We reconstructed the evolution of gene families on a phylogeny for these 10 species, based on aligning amino-acid sequences from single-copy gene families using Muscle v3.6 [37], and constructing coding-sequence nucleotide alignments based on these. Phylogenetic inference was performed using BEAST v.1.6.1 [38] from 10 million Markov Chain Monte Carlo (MCMC) generations under a strict clock model using the SRD [39] model for each gene partition. Three independent MCMC runs converged to the same posterior probability. Birth and death of gene families was inferred under Dollo parsimony using the Dollop program from v3.69 of the Phylip package [40].

To look for potential horizontal gene transfers specifically into the *B. xylophilus* lineage, we used BLASTP to compare predicted *B. xylophilus* protein sequences against the NCBI NR database, producing a candidate set of laterally transferred genes that either had no significant BLAST hit (E-value≤10^−5^) to any nematode sequence or had significant hits only to genes from Aphelenchoidoidea and no other nematode. For each of these candidates, amino acid sequence data was extracted from the NCBI database, aligned using Muscle v3.6 and ML phylogenetic trees estimated using the best-fitting model under the AICc criterion in RaxML v7.2.8. [41]. Clade support was estimated using 100 non-parametric bootstrap replicates in RaxML, and approximately unbiased (AU) statistical tests of tree topology were performed in Consel v1.19 [42].

### Non-coding RNA

The detection and annotation of ncRNA molecules was performed using the LeARN pipeline [43]. This pipeline contains four independent methods: tRNAscanSE for transfer RNA (tRNA) gene detection, NCBI-BLASTN versus a ribosomal RNA (rRNA) sequence database, the Rfam database (release 9.0) to detect common ncRNA families and a mirfold-based pipeline using the mirBase library as a source of micro RNA (miRNA) candidates.

### Repeat analysis

Transposable elements (TEs) in the assembly were identified using two approaches. The first stage consisted of *de novo* identification of repeat families in the assembly based on signatures of transposable elements and assuming fragments of TEs are present throughout the genome. Long terminal repeat (LTR) retrotransposons were identified using LTRharvest which searches for two near-identical copies of an LTR flanked by target site duplications that are close to each other. We also used RepeatModeler (http://www.repeatmasker.org/RepeatModeler.html) which aims to construct repeat consensus from two *de novo* detection programs (RepeatScout and RECON). Repeats present at less than 10 copies in the genome or that were less than 100 bp were excluded from further analysis. The second approach used homology searching of the assembly sequence against curated TEs using TransposonPSI (http://transposonpsi.sourceforge.net/). UCLUST was used to cluster the candidate sequences (with 80% identity) and create a non-redundant library of repeat consensus sequences.

The annotation of repeat candidates involves a search against RepBase and NCBI non-redundant library. Some of these candidates that have some annotations available from program output (for example, from TransposonPSI) were further checked this way. Manual curation of the candidates was carried out to determine coding regions on intact TEs that are potentially active. We found the majority of unannotated elements contained no ORFs longer than 30 bp nor had any significant matches to repetitive elements in the databases.

RepeatMasker (v3.2.8) was used to calculate the distribution of each repeat and its abundance. Custom perl scripts were used to choose the best match from overlapping matches in RepeatMasker output to avoid calculating the same region twice or more when considering repeat content of the genome.

### Annotation of carbohydrate active enzymes

The CAZymes Analysis Toolkit (CAT) [44] was used to detect *B. xylophilus* carbohydrate active enzymes (CAZymes) based on the CAZy database. An annotation method “based on association rules between CAZy families and Pfam domains” was used with an E-value threshold of 0.01, a bitscore threshold of 55 and rule support level 40. The annotation was supplemented and confirmed manually using BLAST search similarities and protein length matches. Expansin-like genes were detected by BLAST search using core modules of known expansin proteins as queries. Putative functions of the proteins were predicted by similarity to known protein modules and presence of catalytic sites using BLASTP search against NCBI's Conserved Domain Database service and InterProScan (www.ebi.ac.uk/Tools/InterProScan).

### Peptidase detection

The MEROPS server was used to identify *B. xylophilus* putative peptidases. The peptidase candidates were derived from MEROPS batch BLAST [45]. The candidates were manually examined in terms of similarity (E-value cutoff 1e-10) to MEROPS proteins and presence of all catalytic sites.

### Effector candidates involved in host-parasite interaction

BLASTP was used to search for *B. xylophilus* homologues of effectors from *M. incognita* [46], *Heterodera glycines* [47] and *Globodera pallida* [48]. An E-value cutoff of 1e-5 was used to identify significant matches. In addition, candidate effectors were sought from the *B. xylophilus* protein set using a bioinformatic approach. Secreted proteins were identified as those having a potential signal peptide at their N-terminus predicted by SignalP 3.0 [49] and no transmembrane domain within the mature peptide as predicted using TMHMM 2.0 [50]. Novel candidate effectors within this secreted protein dataset were identified as those with no BLASTP matches against the NR protein database (E-value cutoff 0.001).

### Embryogenesis

Orthologues of *C. elegans* genes necessary for unequal cell divisions were identified from the *B. xylophilus* protein set by BLASTP search using protein sequences retrieved from WormBase as queries. Their structures were confirmed manually using NCBI and WormBase BLAST.

### RNAi Pathway

Seventy-eight proteins known to be involved in core aspects of the *C. elegans* RNAi pathway were identified from the literature. Protein sequences were retrieved from WormBase (release WS206) and used as queries in TBLASTN and BLASTP searches against the predicted protein and contig databases. All primary BLAST hits returning with a bitscore ≥40 and an E-value≤0.01 were manually translated to amino acid sequence in six reading frames (www.expasy.ch/tools/dna.html), and analysed for identity domain structure by BLASTP (through NCBI's Conserved Domain Database service) and InterProScan. The appropriate reading frame in each case (determined empirically on a case by case basis, but usually that with the largest uninterrupted open reading frame) was then subjected to reciprocal TBLASTN and BLASTP against the *C. elegans* non-redundant nucleotide and protein databases on the NCBI BLAST server (http://www.ncbi.nlm.nih.gov/BLAST), using default settings. The identity of the top-scoring reciprocal BLAST hit was accepted as identity of the relevant primary hit, as long as that identity was also supported by domain structure analysis.

The predicted *B. xylophilus* AGOs were analysed further through annotation of conserved RNase-H-like catalytic residue sites and the MID sub-domain which was then used for a further BLAST search against the *C. elegans* nr protein set (NCBI). The MID domain is highly conserved in functionally comparable AGOs [51], [52].

### Neuropeptide genes

Neuropeptide genes were identified using the BLAST search tool available through the genome consortium website. FLP and NLP search strings were constructed from orthologous *flp* and *nlp* transcript sequences taken from *C. elegans* in the first instance, or another plant-parasitic nematode where a *C. elegans* orthologue was unavailable. Search strings were constructed by concatenating the mature peptides (including basic cleavage sites) encoded by each orthologue. Where this concatenation resulted in a search string of less than 20 characters, the string was repeated end-to-end at least once. Search strings were entered into BLASTP searches of the predicted proteins, and tBLASTn searches of the genome scaffolds, with E-value set to 1000. INS search strings were created by concatenating functionally conserved A and B peptide regions from the *C. elegans ins* gene complement, in addition to a series of mammalian and molluscan orthologues. These were used to search the predicted protein set of *B. xylophilus*, using a higher E-value threshold of 1,000,000. The BLAST returns were annotated for both A and B peptides, which were isolated, concatenated and used as BLAST search strings against the *C. elegans* nr protein set (NCBI) with an increased E-value of 1,000,000. All reciprocal BLAST returns with a Bit score ≥25 and an E-value≤10 were annotated for conserved A and B peptides, and the *C. elegans* orthologue with the highest similarity to these domains was accepted as identity. Putative INS orthologues which did not meet the reciprocal BLAST criteria, but which closely resemble the structure of INS A and B peptide domains are included as putative variant (Var) INS orthologues (≤25, ≥20 bit score; E-value ≥10, ≤20). All hits were analysed by eye for the presence of neuropeptide precursor sequences, dibasic cleavage sites, and analysed for the presence of secretory signal peptides using SignalP 3.0 [49].

### Stress response related genes

Orthologues of *C. elegans* genes were identified from *B. xylophilus* protein set using BLASTP. The families of GSTs, UGTs, CYPs, and ABC transporters were identified with InterProScan. Their structures were confirmed manually with BLAST on NCBI and WormBase.

### Dauer related genes

Protein sequences known to be involved in dauer formation and maintenance were retrieved from WormBase and used as search strings in a series of tBLASTn and BLASTP searches against *B. xylophilus* genome and protein sequences. An E-value cutoff of 1e-10 was used to identify significant matches.

### Chemoreception related genes

Potential chemoreceptors included 7-transmembrane, G-protein coupled receptors (GPCRs), e. g. *Str*, *Sra* and *Srg* gene superfamilies in *C. elegans* [53], and other receptors with transmembrane structures, e. g., gustatory receptors (GURs, orthologues of insect gustatory receptors) in *C. elegans,* ionotropic glutamate receptors (IRs) in *Drosophila melanogaster* [54] and transient receptor potential (TRP) channels [55], [56]. Other putative GPCRs including those for neurotransmitters (e.g., bioorganic amines) and other signal transduction pathways were also searched. BLASTP and InterProScan were used to search for *B. xylophilus* orthologues of these proteins. All primary BLASTP hits returning with an E-value ≤ 0.0001 and coverage ratio ≥ 0.7 (apart from insect GURs, which included only hits of very low similarity to nematode GURs) were analysed for identity and domain structure by BLASTP (NCBI's Conserved Domain Database service), WormBase (WS221) and TMHMM 2.0 [50].

Molecular phylogenetic trees of serpentine receptor and gustatory receptor proteins were built by maximum likelihood method using MEGA5 with the JTT matrix-based model [57]. A few receptors were removed in some families based on very long branch lengths in a preliminary maximum likelihood tree. A maximum likelihood tree with culled proteins was drawn to a scale, with branch lengths measured in the number of substitutions per site. All positions containing gaps and missing data were eliminated.

### Accession Numbers

The contigs resulting from *Bursaphelenchus xylophilus* assembly were deposited in the EMBL/Genbank/DDBJ databases under accession numbers CADV01000001-CADV01010432.

## Results/Discussion

### Sequencing and assembly

Our assembly strategy assembled the 6 pairs of nuclear chromosomes (Figure S2) into 1,231 scaffolds, totaling 74.5 Mb, with half of these nucleotides present in scaffolds of at least 1.16 Mb (Table 1). The size of the assembly is in good agreement with our experimental estimate of 69.0±5.5 Mb (see Text S1, Table S1 in Text S2). The genome size of 74.5 Mb is smaller than that of *C. elegans* and other published nematode genomes except that of *Meloidogyne hapla*. The GC content of the genome was 40.4%, higher than that of other nematodes except for *Pristionchus pacificus*. Most of the mitochondrial genome was assembled in a single 13,410 bp scaffold, and shows a similar gene content and organization to that of *C. elegans* (Figure S3). Analysis of conserved eukaryotic genes (CEGs) showed that 96.77% and 97.98% of CEGs were present as full or partial genes respectively, with an average of 1.08―1.09 genes per CEG (Table 1), suggesting high completeness of the assembly.

**Table 1:** *B. xylophilus* genome general features.

Features	*B. xylophilus*	*M. incognita^a^*	*M. hapla^b^*	*C. elegans^b^*	*P. pacificus^b^*	*B. malayi^b^*
Overall						
Estimated size of genome (Mb)	63–75^C^	47–51	54	100*	?	90–95
Chromosome number	6	Variable	16	6	6	6
Total size of assembled sequence (Mb)	74.6	86	53	100	172.5	95.8
Number of scaffolds/chromosomes	1,231	2,817	1,523^d^	6 chr.	2,894^d^	8,180^d^
N50 of scaffolds (kb)	1,158	83	84^d^		1,244	94^d^
Maximum length of scaffold (kb)	3,612	593	360		5,268	6,534
G+C content (%)	40.4	31.4	27.4	35.4	42.8	30.5
Completeness^e^						
Cegma completeness (%): (complete/partial)	97/98	73/77	95/96	100/100	95/98	95/96
Average CEG gene number: (complete/partial)	1.08/1.09	1.53/1.61	1.07/1.12	1.05/1.06	1.20/1.23	1.07/1.11
Protein-coding regions						
Number of gene models	18,074	19,212	14,420	20,416	21,416	18,348
Number of proteins	18,074	20,365	13,072	24,890	24,217	21,252
Max protein length (amino acids)	7,727	5,970	5,473	18,562	5,054	12,803
Average protein length	345	354	309.7	439.6	331.5	311.9
Gene density (genes per Mb)	242.3	223.4	270	249	140.4	221.8
Mean exon size (bp)	288.9	169	171.5	201.6	96.7	159.8
median exon size (bp)	183	136	145	145	85	138
Mean number of exons per gene	4.5	6.6	6.1	6.5	10.3	5.9
median number of exons per gene	4	5	4	5	8	3
Mean intron size (bp)	153	230	154	320	309	280
median intron size (bp)	69	82	55	66	141	215

a Abad et al [4], b WormBase release 221.

c Genome size was estimated using real time PCR. The genome size of a very closely related species *B. mucronatus* was reported to be 29.8M±0.06 (see Text S1).

d These values are from Opperman et al [5], Dieterich et al [62] and Ghedin et al [92] respectively.

e, Assembly completeness was estimated by CEGs (Core Eukaryotic Genes) with CEGMA software. Assemblies in WormBase release 221 were used for *M. incognita*, *M. hapla*, *C. elegans* and *P. pacificus* genomes.

The genome features of *B. xylophilus*, *M. incognita*, *M. hapla* and *P. pacificus* are based on incomplete genome drafts and represent statistical estimates while C. elegans genome has been completely sequenced and well annotated.

The assembly is approximately 22% repetitive, of which only around 1.3% had characteristics of transposable elements (TEs, Table 2). A complete set of tRNA (Table S2 in Text S2) and rRNA genes were found in the genome. In common with other parasitic nematodes, including *B. malayi* and *M. incognita* [58], SL2 trans-splicing appears not to exist in *Bursaphelenchus,* but we find 25 SL1-like sequences, found in the same tandem repeats as the 5S rRNAs, as in *C. elegans.*

**Table 2:** Summary of repeat families in *B. xylophilus* genome.

Repeat Types	Category	families	num. copies	Coverage	% genome
LINE	LINE	19	119	29,092	0.04%
			(24)	(20,108)	(0.03%)
LTR retroelelment	LTR	103	2,695	567,586	0.76%
			(142)	(161,208)	(0.22%)
TIR+Helitron+mu+mariner	DNA	79	1,421	358,534	0.48%
			(539)	(196,901)	(0.26%)
no TE feature		411	93,885	15,950,464	21.39%
			(25,789)	(5,795,724)	(7.77%)
**Total**		620	98,120	16,905,676	22.46%
			(26,494)	(6,173,941)	(8.28%)

Values in parentheses correspond to the numbers with hits at least 80% length of consensus sequences.

**Figure 3:**
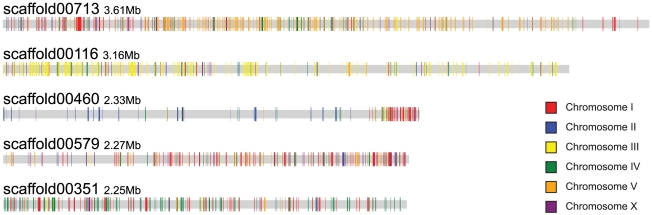
Scaffolds of *B. xylophilus* display a macrosyntenic relationship with chromosomes of *C. elegans*. Orthologues of *C. elegans* genes are highlighted on the five largest *B. xylophilus* scaffolds, coloured according to the *C. elegans* chromosome on which they are found. Scaffolds tend to have a predominance of orthologues from a particular *C. elegans* chromosome, e.g. chromosome V for scaffold00713 and chromosome III for scaffold00116.

Analysis of chromosomal rearrangements between *B. xylophilus* and *C. elegans* identified a similar pattern of macrosynteny to that found between the more distantly related *Trichinella spiralis* and *C. elegans* [59]. Large *B. xylophilus* scaffolds largely contain genes orthologous to those from a single *C. elegans* chromosome. These genes are, however, interspersed by genes orthologous to those from other chromosomes (see Text S1, Figure 3).

### Protein-coding genes and gene families

A total of 18,074 protein coding genes were predicted in the assembly (Table 1). This is fewer than the 20,416 in *C. elegans* (WormBase WS221) and 19,212 in *M. incognita*, although it is higher than the number for *M. hapla*. The average protein length is similar to that of other nematodes, but *B. xylophilus* displays the largest average exon size (289 bp) and the smallest average number of exons per gene (4.5). The *Bursaphelenchus* genome shows a number of characteristics of compact parasite genomes, for example having relatively few, short introns like *M. hapla*, but has a similar repetitive element content to other published nematode genomes, and is overall only slightly smaller.

**Figure 4:**
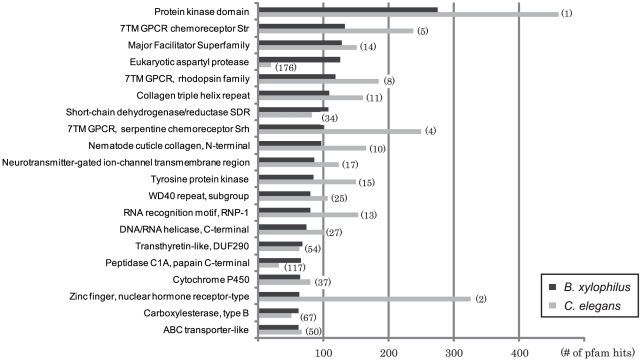
The most frequent Pfam domains found in *B. xylophilus* compared with those in *C. elegans*. Hits with E-value score better than 1e-5 were retrieved and counted. Numbers in parentheses indicate the frequency rankings of each domain in *C. elegans*.

Our automated annotation of the *B. xylophilus* proteome assigned some functional information to a total of 12,483 proteins (69%) (see Text S1 Figure S4). The top 20 Pfam hits in *B. xylophilus* are shown in Figure 4, and compared with hits in *C. elegans*. As part of our annotation approach, *B. xylophilus* proteins were mapped to pathways defined by KEGG, and pathways that are under- and over-represented in this genome compared to *C. elegans* are shown in Table S3 in Text S2 and are discussed in sections focusing on particular biological features below.

**Figure 5:**
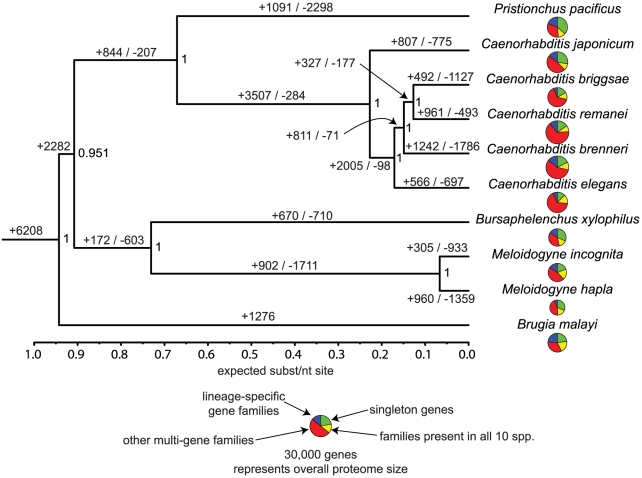
Evolutionary dynamics of gene families in nematodes. Phylogeny is a Bayesian nucleotide phylogeny based on 23 well-aligned single-copy genes present in all 10 species. Values on nodes are Bayesian posterior probabilities. Values on edges represent the inferred numbers of births (+) and deaths (−) of gene families along that edge. Note that our approach cannot distinguish gene family losses from gains on the basal branches of this tree, so for example the value of 1276 gene family gains on the *Brugia* lineage will include gene families lost on the branch leading to the other 9 spp., and similarly the 2282 gains on this branch will include *Brugia-*specific gene family losses. Pie charts in the centre represent the gene family composition of each genome – the area of the circle is proportional to the predicted proteome size, and the four wedges represent the relative numbers of proteins predicted to be either singleton genes (i.e. not members of any gene family), members of gene families common to all 10 sequenced Rhabditida genomes, and members of gene families present only in a single genome, and members of other gene families, present in between 2 and 9 genomes.

**Figure 6:**
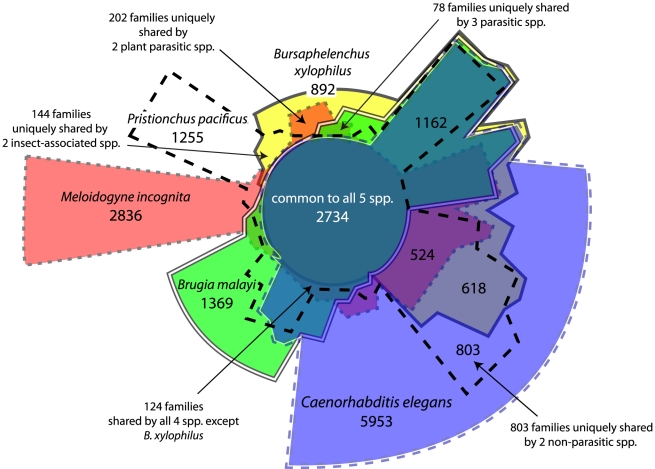
Distribution of orthologous gene families across five selected nematode genomes. Sizes of each region are approximately proportional to the number of genes shared by the overlapping species, which are distinguished by different fill colour and different border styles. The selected genomes represent both taxonomically and ecologically diverse nematodes – *C. elegans* is a free-living bacteriovore, *Brugia malayi* is an animal parasite, *Bursaphelenchus xylophilus* and *Meloidogyne incognita* are plant parasites, while *Pristionchus pacificus* has a close ecological association with a beetle host, but is not parasitic. A phylogeny for these 5 species is shown in Figure 5. A number of overlapping regions are labelled with the number of gene families, as are a number of regions representing comparisons of particular ecological or taxonomic relevance.

The combined predicted proteins from *B. xylophilus* and 9 other nematode species were grouped into 27,547 families of orthologues and an additional 51,942 singleton proteins. We used a molecular phylogeny based on single-copy gene families to reconstruct the distribution and evolutionary dynamics of these gene families (Figure 5), and find that the *B. xylophilus* genome has been relatively conserved over the long divergence from other plant parasitic nematodes. The pattern of sharing of gene families between genomes (Figure 6) shows little obvious phylogenetic pattern, but identifies relatively small numbers of genes - 202 genes shared by the two plant parasitic genomes, and 144 genes shared by *Pristionchus* and *Bursaphelenchus* that both show a close association with insects during their lifecycle – that could be implicated in these particular specializations.

### Cell wall degradation

The plant cell wall is the primary barrier faced by most plant parasites and the production of enzymes able to break down this cell wall is thus of critical importance. A summary of the carbohydrate active enzymes (CAZymes) and expansin-like proteins which may modify plant cell walls detected in *B. xylophilus* and other nematodes is shown in Table 3. *B. xylophilus* contains 34 putative plant cell wall modifying enzymes but compared to other plant parasitic nematodes its composition is unique. Most interestingly, glycoside hydrolase family 45 (GH45) cellulases are present only in *B. xylophilus*. Other plant parasitic nematodes have GH5 proteins that degrade cellulose but no such genes are present in *B. xylophilus.* In addition, GH30 xylanases, GH43 arabinases and GH28 pectinases were also absent in *B. xylophilus*.

**Table 3:** *B. xylophilus* enzymes with predicted plant/fungal cell wall–degrading activities, compared with those in other nematodes.

Substrate	Cellulose		Xylan	Arabinan	Pectin		Other	
Family	GH5	GH45	GH30*	GH43	GH28	PL3	EXPN	Total
*B. xylophilus*	0	11	0	0	0	15	8	34
*M. incognita*	21	0	6	2	2	30	20	81
*M. hapla*	6	0	1	2	2	22	6	39
*C. elegans*	0	0	0	0	0	0	0	0
*P. pacificus*	6	0	0	0	0	0	0	6
*B. malayi*	0	0	0	0	0	0	0	0

Substrate	1,3-glucan	Chitin			
Family	GH16	GH18	GH19	GH20	Total
*B. xylophilus*	6	9	2	7	24
*M. incognita*	0	3	2	2	7
*M. hapla*	0	4	0	1	5
*C. elegans*	0	38	6	5	49
*P. pacificus*	0	9	2	5	16
*B. malayi*	0	16	0	3	19

Cell wall degrading CAZymes found in plant parasitic nematodes are thought to have been acquired via horizontal gene transfer (HGT) because similar genes are absent in almost all other nematodes and because they are most similar to genes from bacteria or fungi [60], [61]. GH5 cellulases have been found in many plant parasitic Tylenchoidea including *Meloidogyne*, *Globodera* and *Heterodera*. Recently, the genome sequence of *P. pacificus* revealed that this nematode also has GH5 cellulases [62]. However phylogenetic analysis suggested that these cellulases were not closely related to those in the Tylenchida and that they are likely to have been acquired independently from different sources [62]. *B. xylophilus* GH45 cellulases have not been found in any other nematode genus and are most similar to those from fungi. Thus these genes have been hypothesised to be acquired via HGT from fungi [11]. In a phylogenetic analysis all 11 GH45 proteins found in the *B. xylophilus* genome are grouped in a highly supported monophyletic group and embedded within a clade of fungal homologues (Figure S5). This supports the idea of HGT from fungi with subsequent duplication within the *B. xylophilus* genome. Recent analysis has revealed that distribution of GH45 proteins is limited to the genus *Bursaphelenchus* and its sister genus *Aphelenchoides* (T Kikuchi unpublished results). The absence of GH5 genes in the *B. xylophilus* genome and the absence of GH45 proteins in Tylenchida nematodes support these hypotheses and suggest that HGT events have repeatedly played important roles in the evolution of plant parasitism in nematodes.

A systematic evolutionary study of plant cell wall modifying genes in Tylenchoidea concluded that those genes were acquired by multiple HGT events from bacteria closely associated with the ancestors of these nematodes followed by gene duplication [61]. *B. xylophilus* is closely associated with fungi and it is likely that this feeding strategy is ancestral for this group as most *Bursaphelenchus* species are solely fungal feeders.

In addition to plant cell wall degrading enzymes, CAZymes are present in *B. xylophilus* which potentially degrade the fungal cell wall. Chitin is one of the main components of fungal cell walls. Proteins related to chitin degradation were identified in the *B. xylophilus* genome. In comparison to the two *Meloidogyne* species, *P. pacificus* and *B. malayi* the number of chitin-related CAZymes in *B. xylophilus* is increased, likely reflecting its fungal feeding activity (Table 4). *C. elegans* has a much larger number of GH18 proteins than *B. xylophilus*. This may be because those proteins have been used in *C. elegans* for specific biological features such as bacterial feeding. Interestingly, six GH16 proteins which may degrade beta-1,3-glucan, another core component of the fungal cell wall, have been identified in the genome while no homologues have been found in other nematodes (Table 4). Because the *B. xylophilus* GH16 β-1,3-glucanase genes are most similar to those from bacteria, it has been suggested that they were acquired from bacteria which were closely associated with its ancestor [63]. This suggests that HGT processes, similar to those associated with plant cell wall modifying enzymes of other parasitic nematodes, have enhanced the ability of *Bursaphelenchus* spp. to feed on fungi.

**Table 4:** CAZyme families with substantial expansions in *B. xylophilus* other than those having putative cell wall degrading process.

Family	GH27	GH109*	GT43
*B. xylophilus*	3	7	12
*M. incognita*	1	1	1
*M. hapla*	0	1	0
*C. elegans*	1	0	7
*P. pacificus*	1	0	3
*B. malayi*	1	0	3

*Although searching by CAT categorized these proteins as GH109, further sequence alignment revealed these were most similar to trans-1,2-dihydrobenzene-1,2-diol dehydrogenases.

Not all of the carbohydrate-active enzymes over-represented in the *B. xylophilus* genome are involved in cell-wall degradation – several other CAZyme families are substantially expanded in *B. xylophilus* compared to other nematodes sequenced to date (Table 4). For example, the genome also has more glycosyl transferases family 43 (GT43) genes than other nematodes. These proteins may have beta-glucuronyltransferase activities, but the reason for the increase in these genes in *B. xylophilus* remains unclear.

### Peptidases and lysosome activity

Peptidases (proteases) catalyse the cleavage of peptide bonds within proteins, play important functions in all cellular organisms and are involved in a broad range of biological processes. In nematodes, peptidases play critical roles not only in physiological processes including embryogenesis and cuticle remodeling during larval development but also in parasitic processes such as tissue penetration, digestion of host tissue for nutrition and evasion of the host immune response.

In our analysis 581 peptidase genes were identified in *B. xylophilus*, which is the largest number in any characterized nematode genome (Table 5), with peptidase families involved in extracellular digestion and lysosomal activities particularly expanded (see Text S1, Table S4 in Text S2). One family of endopeptidases appears to have been acquired by HGT from an ascomycete fungus (Table 6). In addition, *B. xylophilus* contains an expanded number of GH27 proteins homologous to the *gana-1* gene of *C. elegans* (Table 4), which has α-galactosidase and α-N-acetylgalactosaminidase activities and is localized to lysosomes [64].

**Table 5:** Summary of peptidases in *B. xylophilus* and other nematodes.

	*B. xylophilus*	*M. incognita*	*M. hapla*	*C. elegans* *	*P. pacificus*	*B. malayi*
Asp	73 (39)	14 (9)	11 (6)	24 (20)	35 (22)	17 (14)
Cys	142 (108)	100 (59)	55 (35)	123 (97)	88 (50)	126 (66)
Metal	190 (164)	135 (115)	89 (71)	181 (171)	184 (152)	142 (108)
Ser	155 (86)	85 (45)	53 (22)	136 (110)	166 (83)	99 (51)
Thr	20 (16)	28 (18)	47 (16)	22 (19)	20 (16)	25 (19)
Unassigned	1 (−)	1 (−)	1 (−)	1 (−)	1 (−)	3 (−)
Total	581 (414)	363 (247)	256 (151)	487 (418)	494 (324)	412 (261)

According to Merops, the proteins classified by catalytic types: aspartic (Asp), cystein (Cys), metallo (Metal), serine (Ser) and threonin (Thr). Numbers of proteins in which all conserved catalytic residues found are shown in parentheses.

*Isoforms from one gene were counted only once.

**Table 6:** Putatively laterally transferred genes, supported by phylogenetic evidence.

Protein ID	Annotation	Likely source
BUX_s00460.56BUX_s01281.77BUX_s01281.82BUX_s00110.147	Aspartic-type endopeptidase	Ascomycete fungiAscomycete fungiParamecium/Ascomycete fungiAscomycete fungi
BUX_s01337.17	6-phosphogluconolactonase	Bacteria – firmicutes – *Paenibacillus*
BUX_s00351.346	Contains cystatin-like domain	Gamma-proteobacteria
BUX_s01281.503	HAD-superfamily hydrolase	Bacteria – firmicutes
BUX_s00119.43BUX_s01147.110BUX_s00397.15BUX_s00119.44BUX_s00397.16BUX_s01116.1BUX_s01038.221BUX_s01288.37BUX_s00397.6BUX_s00036.112BUX_s00036.113	GH45 hydrolase	Ascomycete fungi
BUX_s01066.145BUX_s01066.63BUX_s01066.142BUX_s01066.65BUX_s01066.144BUX_s00705.10	GH16 β-1,3-glucanase	Gamma-proteobacteria

Likely source indicates sister-taxon or sister-taxa of the gene copy on the phylogeny. Annotation is either interPro or GO term annotation for the *Bursaphelenchus* gene. Consecutive boxes of similar shading indicate genes within a single orthoMCL family that are likely to have diversified from a single laterally transferred ancestral copy.

The gut granules of intestinal cells in *C. elegans* are intestine-specific secondary lysosomes, so lysosomal enzymes play important roles in the digestion of food proteins in nematodes. *B. xylophilus* has an expanded repertoire of peptidases and other digestive enzymes that are either secreted or localised in lysosomes and so may play important roles in food digestion. Genes in the lysosome pathway were the most significantly over-represented in *B. xylophilus* (Table S3 in Text S2). *B. xylophilus* uses food sources such as fungi and woody plants that may be difficult to digest and the expansion of digestive peptidases in the nematode is therefore consistent with its unusual life style.

### Effector candidates

Plant parasitic nematodes produce a variety of secreted proteins that mediate interactions with their hosts – these encompass a variety of functions and include the cell-wall modifying enzymes discussed above. Such proteins have variously been termed “parasitism genes” or “effectors” and encompass any protein secreted by the nematode into the host that manipulates the host to the benefit of the nematode. For example, cyst nematodes produce effectors that mimic plant peptides and which may help initiate the formation of the biotrophic feeding structures induced by these nematodes [65], as well as proteins that suppress host defence responses [66]. We found that the majority of effectors from other plant parasitic nematodes have no homologues in *B. xylophilus.* Some significant matches to effectors from all three species were found (Table 7). However, the *B. xylophilus* sequences that matched these effectors, except cell wall degrading enzymes, either did not have predicted signal peptides or, if a signal peptide was predicted, homologues were also present in a wide range of other species including *C. elegans* and animal parasitic nematodes. Both these lines of evidence suggest that the *B. xylophilus* homologues identified in this analysis are not true effectors that play a role in parasitism. These findings are consistent with the differing biology of the various nematode groups; root knot and cyst nematodes are biotrophic species whereas *B. xylophilus* is a migratory endoparasite that does not rely on biotrophy.

**Table 7:** *B. xylophilus* effector candidates.

CN/RKN gene	*B. xylophilus* match*	Signal peptide?	Other matches
*M. incognita* AF531162	116.149	Yes	Secreted ground-like domain proteins from *Brugia malayi, Caenorhabditis spp.*
*M. incognita* AY135362	1063.147	No	Leukocyte cell derived chemotaxin, *C. elegans* hypothetical proteins
*M. incognita* AY135364	579.238	Yes	*Caenorhabditis spp.* hypothetical proteins
*M. incognita* AY135365	116.427	No	Various nematode failed axon connection proteins, Glutathione S transferase-like domains
*M. incognita* AY134440	460.216	Yes	Histidine acid phosphatase family proteins from various nematodes.
*G. pallida* SPRYSECs	294.74, 1281.146, 1066.75	No	Various SPRY domain containing proteins from a range of organisms
*H. glycines* hg-sec-12	110.167	Yes	Regulator of innate immunity, inhibitor of metalloproteinase
*H. glycines* 30G12	1198.164	No	Pioneer
*H. glycines* G8A07	1254.197	Yes	*Caenorhabditis spp.* hypothetical proteins, MFS transporter domain
*H. glycines* G16H02	1513.371	Yes	Secreted hypothetical proteins from *Caenorhabditis spp.*
*H. glycines* hg-sec-6	713.186	Yes	Secreted selenoproteins from a range of nematode species
*H. glycines* hg-sec-8	1653.98	Yes	Endoplasmin precursor from a wide range of species
*H. glycines* hg-sec-3	298.203	No	Protein disuphide isomerase
*H. glycines* SCN1120	705.53	Yes	E3 Ubiquitin ligase, other matches also with signal peptide
*G. rostochiensis* VAP-1	1653.397	Yes	Venom allergen proteins from a range of nematodes
*H. glycines* hg-sec-11	351.339	Yes	Transthyretin-related proteins from a range of nematodes
*H. glycines* G33E05	1281.39	No	Pioneer
*H. glycines* G10A06	1254.237	No	RING finger proteins from a wide range of species
*H. glycines* G8H07	116.506	No	SKP1 proteins from a wide range of species
*H. glycines* AF345800_1	347.17	No	GTPase, TiTiN proteins from a wide range of species

*B. xylophilus protein IDs are shown in abbreviated styles (e.g. “116.149” represents the protein name “BUX_s00116.149”)

CN, cyst nematode; RKN, root knot nematode.

There are two possible exceptions. *B. xylophilus* contains homologues of venom allergen proteins. These proteins are present in all nematodes investigated to date and are thought to be important for the parasitic process of animal and plant parasites (*e.g.* [67], [68]). Several venom allergen proteins from *B. xylophilus* have been characterized and are known to be expressed in the oesophageal gland cells [69]. Our HGT analysis identified a putative cystatin, or cystein protease inhibitor, apparently acquired from a bacterium (Table 6). Cystatins are well known as immunomodulatory pathogenicity factors in the animal parasitic filarial nematodes [70], so this protein could potentially play a role in parasite-host interaction in a plant parasitic nematode. However proteins from this family are involved in regulating a variety of endogenous proteinase activities in many cellular roles and, for example, are described as having anti-fungal properties [71], so the function of this protein will require experimental verification. We also identified a total of 923 predicted secreted proteins in the *B. xylophilus* genome that show no significant similarity to proteins from other species (Dataset S1), representing a pool of candidates that may play a role in the interaction between *B. xylophilus* and the other organisms with which it interacts. Very few (5) of these sequences produce matches against other plant parasitic nematode ESTs, consistent with previous studies which have shown that secreted proteins of parasitic nematodes often bear a high proportion of novel genes [72].

### Utilisation of host secondary metabolites

Detoxification of potentially damaging compounds is an important process for any organism to cope with its environment and may be particularly crucial for parasitic organisms, which come under attack from host responses to infection. In particular, plant parasites must cope with a wide range of secondary metabolites that plants generate in order to protect their tissues [73].

*B. xylophilus* principally inhabits the resin canals of its pine hosts. The resin to which it is exposed – a complex mixture of compounds, including terpenoids [74] and cyclic aromatic compounds [75] – is likely to have nematocidal activity and, like the detoxification of xenobiotics by *C. elegans* [76], would be expected to proceed in three distinct phases: (I) the addition of functional groups to molecules, making them more suitable substrates for downstream; (II) the actual detoxification reactions; and (III) efflux. Cytochrome P450s (CYPs) represent the most important group of phase I proteins, and *B. xylophilus* encodes a similar number of CYPs to that found in *C. elegans* (Table 8). Of the two main families of phase II detoxification enzymes – the glutathione S-transferases (GSTs) and UDP-glucuronosyl transferases (UGTs), we identified 41 full-length and 26 partial GSTs, and 60 UGTs, similar numbers to those found in *C. elegans* (Table 8, Figure S6). The final phase of the detoxification process involves ATP-binding cassette (ABC) transporters actively exporting detoxified xenobiotics. A total of 106 ABC transporters were detected in the *B. xylophilus* genome; this number was about twice that for *C. elegans* and about three times that for *M. incognita* (Table 8), suggesting that *B. xylophilus* is particularly enriched in genes responsible for the efflux of detoxified molecules.

**Table 8:** Main phase I, II, and III detoxification enzymes in *B. xylophilus* and other nematodes.

		*B. xylophilus*	*M. incognita* ^b^	*M. hapla*	*C. elegans* ^a^	*P. pacificus^c^*	*B. malayi*
Phase I	CYP450	76	27	12*	81	198	5*
Phase II	GST	67	5	4*	68	54	5*
	UGT	60	68^*^	26*	65	139	2*
Phase III	ABC	106	36^*^	17*	60	129	46*

a WormBase (WS211), b Adad et. al [4], c Dieterich et. al [62], *numbers are based on pfam search (e-value<1e-5).

Finally, we investigated genes involved in regulating the detoxification process. In *C. elegans*, the transcription factor SKN-1 regulates expression of many detoxification enzymes [77], and SKN-1 activity is in turn controlled by a number of different pathways (see Figure S7). In the presence of oxidative stress or electrophilic compounds, SKN-1 induces the expression of many phase II detoxification enzymes. Orthologues of all these regulatory pathways can be identified in *B. xylophilus*, suggesting that the regulation of xenobiotic degradation may be conserved in nematodes.

There are other signs that *Bursaphelenchus* may have an unusual repertoire of genes involved in the defence against or utilisation of complex pine tree metabolites – in our KEGG pathway analysis, xenobiotic and drug metabolism through CYPs were among the pathways showing most significant enrichment in gene copy number over *C. elegans*, confirming that other genes, likely to be involved downstream of the CYP genes themselves, as well as efflux effectors, are notably enriched (Table S3 in Text S2). Furthermore, our search for carbohydrate active enzymes reported a number of genes classified into the GH109 family of glycosyl hydrolases (Table 5) that on closer inspection proved to be most similar (approx 39% identity and E-values<1E-50) to enzymes displaying trans-1,2-dihydrobenzene-1,2-diol dehydrogenase activity, which is involved in the pathway downstream of cytochrome P450 in the metabolism of naphthalene and other polycyclic aromatic hydrocarbons (PAHs) [78] (Table S3 in Text S2). While PAHs, including naphthalene itself, are known to be produced in small quantities by a few plant species [79], they are not known from pines, and it seems more likely that this enzyme is homologous to naphthalene-degrading enzymes but acts on some of the many other aromatic molecules generated by plants [73].

It seems likely that *B. xylophilus* has a larger number of detoxification enzymes than other plant parasitic nematodes (*M. incognita* and *M. hapla*), with similar or expanded repertoires of such genes to those reported for the free-living *C. elegans* and the necromenic *P. pacificus* [62] for the various components of the detoxification process. This expansion in detoxification process components may reflect the variety of stressful environments that it encounters during its life cycle, and perhaps the particular challenges of inhabiting living tissues in a plant host that produces diverse secondary toxic metabolites.

### Development

*B. xylophilus* embryos seem to form the anterior-posterior axis quite differently from those of *C. elegans* as the point of sperm entry becomes the future anterior end of the animal [80]. Surprisingly, however, other early events in *B. xylophilus* embryos, such as pronuclear meeting and posterior spindle movement followed by the unequal first cell division are quite similar [80]. Therefore, it is informative to compare and contrast the proteins that control these processes in these two species. Orthologues of the majority of *C. elegans* proteins involved in these processes were identified in *B. xylophilus* and appear to be highly conserved. However one putative homologue of the serine/threonine kinase protein PAR-1 was quite distinct in *B. xylophilus* from that in *C. elegans* in that the former was considerably smaller (467 AA compared to 1,192 AA in *C. elegans*); the implications of this difference are unknown.

The formation of dauer (or infective) larvae specialized for surviving adverse conditions or for invading host organisms is an important life stage for many nematodes. In *B. xylophilus,* we identified orthologues of most genes involved in pathways which regulate dauer larva formation and recovery in *C. elegans* [81] (Table S5 in Text S2). We also identified orthologues of genes involved in *C. elegans* dauer pheromone synthesis (see Text S1). As *C. elegans*, adverse conditions trigger *B. xylophilus* to enter the third-stage dauer larva (DL3 or dispersal third-stage larva LIII) (Figure 2). Pathways that respond to these environmental cues may be more conserved in *B. xylophilus* than in other parasitic nematodes, most of which use different cues when forming a dauer (infective) stage. In addition to DL3, *B. xylophilus* has a specialized stage called the fourth-stage dispersal larva (DL4 or LIV). *B. xylophilus* DL3 develop into the DL4 when stimulated by the presence of the vector beetle *Monochamus alternatus* and become ready to board the vector [82], [83]. Previous studies showed that several novel genes are expressed specifically in the DL4 nematodes [10], suggesting that *B. xylophilus* responds to different environmental stimuli, and likely uses distinct pathways and proteins to control this part of the lifecycle.

### Neuropeptide-encoding genes

Nematode neuropeptides are encoded on *flp* (FMRFamide-like peptide), *nlp* (neuropeptide-like protein) or *ins* (insulin-like peptide) genes [84], [85]. Diverse arrays of neuropeptides exist within every nematode species that has been studied, and neuropeptide receptors are promising potential drug targets [86]. The complexity of this peptidergic signalling environment likely aids behavioural diversity and plasticity in spite of the structurally simple nematode nervous system. We find *B. xylophilus*'*s flp* and *nlp* gene complements are typical of those seen in other parasitic nematode species [87], [88], although the absence of two *flp* genes - *flp-30* and *-31* - is noteworthy (Table S6 in Text S2), as these genes have previously been considered unique to *Meloidogyne* spp. [4], [87]. Their absence from *B. xylophilus* suggests they may associate with an obligate parasitic lifestyle. The discovery of seven *ins*-like orthologues in the *B. xylophilus* genome is significant as the first description of nematode INS-like peptides outside *C. elegans* (Table S6 in Text S2, Dataset S2).

### Chemoreception

Chemoreception governs essential aspects of the life of many invertebrates, including the search for mates and hosts and the timing of critical steps in their life cycles. Chemoreceptors constitute one interface between the animal and its world, and could be expected to exhibit local adaptations to the specific chemosensory niche of each organism. The main group of putative chemosensory genes in nematodes is represented by serpentine receptors, which are GPCRs, include a large number of families and are also important drug targets. We find representatives of most *C. elegans* serpentine receptor families in the *B. xylophilus* genome, but many represent specific expansions, so the two species have related but largely distinct repertoires. The total number of serpentine receptor genes identified from *B. xylophilus* represents only 10–20% of the number found in *C. elegans* [53] but 35–45 times of those in *M. hapla* [5]. It is unclear whether these striking differences represent a reduced and/or expanded chemosensory systems in various nematodes, or whether additional gene families have been expanded to cover some of the chemosensory spectrum in *B. xylophilus* and other species. Other chemosensory genes identified include gustatory receptors, GPCR receptors for a range of neurotransmitters that could have chemosensory roles, and members of the ionotropic glutamate receptor family [54], among others (see Text S1, Figure S8–S10).

### RNAi pathway

The *B. xylophilus* genome encodes more predicted orthologues of *C. elegans* RNAi pathway effectors (37 of a potential 78) than found in *M. incognita* (27) and *M. hapla* (28) (unpublished data). Whilst *B. xylophilus* has orthologues of eight of the nine small RNA biosynthetic protein-encoding genes considered, dsRNA uptake and spreading genes are not well represented, e.g. no *sid* gene orthologues were identified with *rsd-3* the only representative gene identified. RNA-dependent RNA polymerases (RdRps) are expanded relative to *C. elegans*, with four *ego-1*-, two *rrf-1*-, and three *rrf-3*-like orthologues. Sixteen Argonaute (AGO) genes were identified relative to the 27 of *C. elegans* and, for some of these, there was divergence within the catalytic and RNA-binding MID subdomains; other RNA-induced silencing complex (RISC) cofactors were identified (*ain-1, tsn-1* and *vig-1*). Whilst the short interfering RNA (siRNA) inhibitor *eri-1* was not found, microRNA (miRNA) inhibitors (*somi-1*; *xrn-2*) were identified. Nuclear effectors were reasonably well represented such that most of the components of a functional RNAi pathway were identified within the *B. xylophilus* genome. See Text S1 and Table S7-S11 in Text S2 for details.

### Concluding remarks

In addition to its status as an economically important plant pathogen, *B. xylophilus* is remarkable for its unusual biological traits that relate to its complex ecology. During its life cycle it occupies two distinct habitats – an insect and a tree – where it exploits a number of different food sources, including plant tissues and a wide variety of fungi. This adaptability to a number of different niches is reflected in its genome sequence. The presence of a rich repertoire of detoxification enzymes and transporters reflects *B. xylophilus*' habitat in the resin canals of its host trees, where it is exposed to a cocktail of secondary metabolites, and its elaboration of a narrow subset of carbohydrate metabolizing enzymes reflects it adaptation to break down plant cell walls. The unique complement of genes involved in cellulose degradation, and other catabolic enzymes and the absence of effectors previously known to function at the host-parasite interface, confirms that *B. xylophilus* has a mode of parasitism that is distinct from other plant parasitic nematodes. This parasitism is mediated by a unique suite of parasitism-related genes, assembled through a combination of gene duplication and horizontal gene transfer. The genome provides strong evidence of multiple independent horizontal gene transfer events and these have shaped the evolution of this group.

Most importantly the genome sequence will act as a foundation for functional studies using a wide range of techniques and will directly inform efforts aimed at controlling this parasite. The identification of genes involved in nematode invasion and feeding from the plant will empower efforts to understand the interaction of *B. xylophilus*'s with its host. One exciting possibility is the potential for genomic information from the hosts of *B. xylophilus* to facilitate understanding of the host-parasite interaction and associated pathology; host genetics is likely to play a key role in the disease as *B. xylophilus* is non-pathogenic to American pine species. We have identified genes involved in a range of crucial biological processes, many of which, such as neuropeptides, GPCRs and developmental genes could be viable control targets. The presence of a rich set of RNAi pathway effector genes gives much hope that reverse genetics will underpin future functional genomics efforts in this species. In this way, the genome sequence provides the opportunity to identify and validate putative control targets without the need to rely on *C. elegans* and make assumptions on conserved functionality/importance between nematodes from different clades.

To our knowledge, only seven nematode genomes have previously been published, and data are available for only a handful more, mostly from the genus *Caenorhabditis*. Given the breadth of the nematode phylum, genomic information from any new nematode species is an important advance but, *B. xylophilus*, in particular, is the first species to be sequenced from clade 10, and the first from the order Aphelenchoidea. We hope that with the other imminent nematode genomes being sequenced, *B. xylophilus* will serve as an important comparator. These data provide a rich resource for those trying to develop novel control strategies directed against *B. xylophilus*. In addition, the parasite's unusual life cycle makes this genome sequence a unique resource to investigate the association between genome structure and lifestyle, casting new light on the many conserved processes for which the free-living non-parasitic *C. elegans* remains the pre-eminent model.

## Supporting Information

Dataset S1 *B. xylophilus* novel putative secreted proteins.
(DOC)

Dataset S2 Amino acid sequences of *B. xylophilus* INS-like proteins.
(XLS)

Figure S1 Accuracies of gene predictions by *ab-initio* methods and combined by EVidenceModeler (EVM). Gene prediction accuracy was calculated at the nucleotide, exon, and complete gene level using 200 manually curated gene models as references. Label Augustus, Snap and Genemark.hmm represent *ab-initio* gene predictions by each predictor. EVMall, which is the combined one by EVM with the optimised weight, includes the three *ab-initio* predictions and the GeneWise predictions based on pfam protein similarities. EVMall+PASA includes EVMall and Program to Assemble Spliced Alignments (PASA) alignment assemblies and corresponding terminal exon supplement. Sn, sensitivity.
(PDF)

Figure S2 Chromosome numbers of *B. xylophilus* and *B. mucronatus. B. xylophilus* (Ka4C1 line) chromosome was observed to be 2n = 12. Bar = 2 µm.
(PDF)

Figure S3 Gene orders of mitochondrial DNAs of *B. xylophilus* and *C. elegans.* Arrows indicate the direction of transcription of genes. The genomes contain 12 protein-coding genes (atp6, cob, cox1-3, nad1-6 and 4L), two rRNA genes (rrnS and rrnL), tRNA genes (circles with one letter codes to indicate transferred amino acid) and non-coding region (AT). Note that the *B. xylophilus* mtDNA sequence is not completed, lacking a short AT rich sequence.
(PDF)

Figure S4 Distribution of the three major gene ontology categories assigned to *B. xylophilus.* Predicted genes can have more than one GO term. The x axis indicates the percentage of the term compared to the total of the terms.
(PDF)

Figure S5 Unrooted phylogenetic tree of GH45 proteins. Amino acid sequences of GH45 proteins in CAZy website (www.cazy.org) were retrieved from uniprot. The maximum likelihood phylogenetic tree was made using Phylogeny.fr (www.phylogeny.fr) using default setting. Proteins with short lengths or with long branches in the preliminary tree were removed from analysis.
(PDF)

Figure S6 GST classifications in *B. xylophilus* and *C. elegans*. Full-length GSTs containing both conserved N and C domains are classified into the classes. GST phylogeny tree is reconstructed according to Zimniak & Singh [91].
(PDF)

Figure S7 Signalling pathways involved in phase II enzyme expression. The XREP-1/DDB-1/CUL-4 complex (red), the DAF-2 pathway (green), and glycogen synthase kinase (yellow) negatively regulate SKN-1 (dark blue), whereas the p38 MAPK pathway (light blue) positively regulates SKN-1. The relationships among these pathways are not clear. When animals are exposed to xenobiotics, SKN-1 accumulates in the nucleus and induces the expression of many phase II enzymes. *C. elegans* protein names are followed by more general names in brackets.
(PDF)

Figure S8 Phylogenetic tree of 16 gustatory receptors. The phylogenetic tree was built by maximum likelihood method according to MEGA5 based on the JTT matrix-based model with uniform rate. Three proteins of *B. xylophilus*, (GUR-1, -2 and -3 indicated with bold) proteins of nematodes *C. elegans*, *C. remanei*, *C. briggsae* (GUR indicated with red), and proteins of insects *Drosophila pseudoobscura*, *Anopheles gambiae*, *Culex quinquefasciatus*, *Tribolium castaneum* (GR with blu) collected from NCBI protein database were used in the analysis. The scale bar indicates number of amino acid changes per site. Bootstrap values more than 50% form 1000 replications were shown on appropriate branches.
(PDF)

Figure S9 Maximum likelihood tree of STR superfamily chemoreceptor proteins in *B. xylophilus*. The phylogenetic tree was built by maximum likelihood using MEGA5. All 5 families (Str, Srd, Srh, Sri and Srj) from Str superfamilies of *B. xylophilus* were included in the tree. The scale bar indicates number of amino acid changes per site. Bootstrap values more than 50% were shown in the tree.
(PDF)

Figure S10 Maximum likelihood tree of SRT family chemoreceptor proteins in *B. xylophilus* and *C. elegans.* The phylogenetic tree was built using MEGA5. The scale bar indicates number of amino acid changes per site. Bootstrap values more than 50% were shown in the tree.
(PDF)

Text S1 Supplementary text – Detailed Result.
(DOC)

Text S2 Supplementary tables.
(DOC)
