# Supplementary material for: Genomic Insights into the Origin of Parasitism in the Emerging Plant Pathogen Bursaphelenchus xylophilus
Source: PLoS Pathog. 2011 Sep 1;7(9):e1002219. doi: 10.1371/journal.ppat.1002219 (PMC3164644; doi:10.1371/journal.ppat.1002219)
Supplement: Dataset S1 — B. xylophilus novel putative secreted proteins. (DOC) [file ppat.1002219.s001.doc]

**Supplementary File.** *Bursaphelenchus xylophilus* neuropeptide sequences. **a. Insulin-like (INS) precursors.** A peptides are highlighted in red, B peptides are highlighted in yellow; note the presence of two *ins-1* sequences (*ins-1a* and *-1b*), and variant INS peptides (denoted ‘var’) with similarity to *ins-17* and *-32*. **b. FMRFamide-like peptide (FLP) precursors and** **c. Neuropeptide-like peptide (NLP) precursors.** Peptide-encoding regions are denoted by yellow highlighting, with basic cleavage recognition sites underlined and putative mature peptides in bold text; secretory signal peptides are denoted by bold, underlined, italic text; * denotes stop signal.

**a.**

**>*Bx-ins-1a*_[BUX_s01038.98]**

***MEFKLTFSLTTLALLLTVFLSQSSTASA***SFRLCGFKLTTTLQHLCKHKTCGGYQLHTRKRSIASFLDEISAEAAALVDERPAEEVDEHPLRPLLADLRIRKRSGIATECCEKRCNLAYLRTYCCAGFEA*

***>Bx-ins-1b*_[BUX_s00116.474]**

***MTTHMKPLFPVLILVQLLLFRYADA***NFRLCGFKLTTTLQHVCKHQVCGGYQVQTQKRALSDYNNPFFRFRIMGYKRKSHQKRSGVATQCCQNRCSLSYLRTYCCAGFER*

***>Bx-ins-6*_[BUX_s01038.83]**

***MTTDIAASATQKSARIQRNCGGSWRPSVSKRTLIIVLCLLLSTHGLASA***ETVRICGEKLIKKVQALCTFIVDGATHVCFKSMKNHVSRRHIFLEHGHHRLNKRSGIADDCCVNRCTIEHMKRVCCDDEELDKYVKQRLERR*

***>Bx-ins-17var*_[BUX_s00036.210]**

***MAFKLALTVLLLVLATLEA***KKYCGGQFNQLQKKVCTYDKQDSPCLGGPHLNREIQDKCCKEGCSLGDISKTCCFTDSCLKSCYPGLEHQTGKKRINKMGNVY*

***>Bx-ins-18*_[BUX_s00333.44]**

***MERLVLFLWLVVLCNG***QIPTEFPEDRLPAFHFCPAGGQTFVEALMVACPLRKKRDLKSKRATINPQSMYSLMLSKSRGWNKPAYDEFADICCKFGCRYEDFTQYCNPF*

***>Bx-ins-31*_[BUX_s00466.37]**

***MKLLFFLALFLAVLGLVIA***EGPRRTCGDKLIARVKEVCESGKRLKLMSDVSQDCCAKPCT

DEQIKENHCTPKE*

***>Bx-ins-32var*_[BUX_s00466.38]**

***MKCEFGVIALLVLTAVFALLSLGLA***DDDTEQRRTCGGKLSSRICEACSGCPCSLDPKRLRLLDDGDLPTKCCSVKCSNEEIRAVVCPDHPTCP*

**b.**

**>*Bx-flp-1*_[BUX_s01145.42]**

***MTPHGAGVLLGILTVIYVKIGVEG***CSGTGAFCRFYSQLDVMDQALINELMEEVQHAKPQKRDSKFTGEFGKK**GSEPNFLRFG**KR**AAPAPNAAGANFLRFG**K**SGADPNFLRFG**KR**ATEFRLDATEPNFLRFG**KR**PDPSAMSNNFLRFG**KR**SSLDTLDREQRQSNNFLRFG***

**>*Bx-flp-2*_[BUX_s00351.133]**

**MTYALSLMLVVVLVMEMCHS**VPEQDFYRSENSNAMPLYMERR**FSKFRGEPVRFG**KR**AYREPIRFG**KRAYMPNNQISNGGYGQNAEN*

**>*Bx-flp-3_*[BUX_s00351.132]**

**MSGWTVTFFALFLVGCTVA**EKTTEDQTAEKRAHPDGQMLRASNWISTRPMMSPDNEIGYLIQYLEKR**ASSMENDAESRDIRSPLGTMRFG**KR**ADGPLGTMRFG**KR**NPLGTMRFG**KR**NPLGTMRFG**KR**AEGPLGTMRFG**KRTPDFYDY*

**>*Bx-flp-4_*[scaffold00139:282497..282919]**

***MGKKALSSLTALRIFLVVEYPSCC***RKKGAKTFWNWCVDLGKLSRINSRKSMQILKQLCGSYLRHFLDKIQFILIHFTVQKFNSPHFQKRENILKALYRLNDQNSYRLYRASR**SNGKPTFIRFG**KRSPPAYDGYEHADFNTQ

**>*Bx-flp-5*_[scaffold01144:14299..14201]**

R**EARAPKAKFIRFG**R**AGQKFIRFG**RRSAFNPLLKFRRNSVSAKKFLRSAK**QKISKFIRYG**KLDSFTMKFGKGSGKKFLRFRYLR**EARAPKAKFIRFG**

**>*Bx-flp-6*_[BUX_s01259.10]**

**MRHSLVLVALATVIAAEFAVA**VTDLCDQFPDLAGCIKPEQVKR**KSAYMRFG**KRSLSLGEPVEEDDAEPFAAYEKR**KSAYMRFG**KRSGGMLEVPEDDGVEMEKR**KSAYMRFG**KR**KSAYMRFG**KRSGQDFLDNSDQPMEMHKRKVAALR*

**>*Bx-flp-7*_[BUX_s00961.59]**

**MLPQFKSLLVPLLLVVFFTGTMA**QDILESYYKR**APMDRASMVRFG**KR**APMDRASMVRFG**KRDSDDFE*

**>*Bx-flp-8*_[BUX_s00422.462]**

**MSSVLPSLLLVVMVGITVG**QKAYPEDELRVCELTDLETLSLSKCELEGRIAELENKIREIEDTVMSQGNHVHVKGEVAKR**KNEFIRFG**KRSLATVLDHMPHSRWNPHSDNALRSLSMVKR**KNEFIRFG***

**>*Bx-flp-11*_[BUX_s00961.21]**

**MKLSALILVLALIVCAVKA**QAEFDEENGYAKR**SMRNALVRFG**R**SGMRNALVRFG**KRSLGNDYAEAKR**AQSAPEPFVRFG**RSAPSHFALYDYYDI*

**>*Bx-flp-12*_[BUX_s00422.464]**

MYVFITARKGELVESNDEMLARIQGQLLNALEMLQVYQEGPETPQVVGISPSQMKFTEKR**RNKFEFIRFG**RR*

**>*Bx-flp-13*_[BUX_s00116.601]**

**MQTSSVLAIVSFLAVLLSCAFA**ETDSVLDYDDLDEVMAKR**AYAGPLIRFG**KR**SMQRALRNSPITDPLIRFG**KRSDVIESKR**PDHSPLIRFG**KR**AAFRSAPHIRFG**KRSDDDALYEW*

**>*Bx-flp-14*_[BUX_s00422.269]**

**MSRVADPSSLLMANTKIIWSLLGFVFVVQVIEA**TVAQTQVEVNCGRILANNNLDNEDKQLLCKIYQQSSSLEQLGAIVSDSLERFMGEATALEEEARPKR**KHEYLRFG**KR**KHEYLRFG**KR**KHEYLRFG**RK*

**>*Bx-flp-16*_[scaffold01281:994016..995015]**

ILQNFKKKFFNKIFFHQKIQKNQKKMFLHPLQYLQLISRHFSGQEQVVESAPEAELPAQKR**AQTFVRFG**KR**AQTFVRFG**KR**AQTFVRFG**

**>*Bx-flp-17*_[BUX_s01145.17]**

**MLGLIIVAGLCSQLAFA**FLTPQQNAKLSELCSANPNLFVCTLAEYLDESMIDIDSLIPAEQRDSLQNPAKNFIGSRMQKR**KSAFVRFG**KRSQGNSEYAPEDLALNFPIDGILNSRPAR**KSSYIRFG***

**>*Bx-flp-18*_[BUX_s01146.1]**

**MSNQWLLVCVMGVVGVCTVQA**AFFRGQPWKELFIADDSRADKRSVAYLPTLDEFSSYGSYYYNSPDEVDGYRQAKR**DDQAMPGVLRFG**KRADIDKK**EMPGVLRFG**KRSDGEAEKK**AVPGVLRFG**KR**SDMPGVLRFG**KR**DGAEMPGVLRFG**KK**SEMPGVLRFG**KR**SDMPGVLRFG**KR**GDMPGVLRFG**RK*

**>*Bx-flp-19*_[BUX_s00579.585]**

MPILSASVESDEPEEYFYIPNPYPSWGVQYPSHAEEPHRMKK**WASSLRFG**KR**GPGWASQVRFG***

**>*Bx-flp-20*_[scaffold01211:229312..229593]**

TALLTSNLLSDTDAVFGEQPADPSFLSRSPYSTLYYANDNPQAQAPAQLLGAEAAIPGQKR**AYMRLG**KR**AYMRLG**KRADQLQEMMEKR**ARLRLG**

**>*Bx-flp-21_*[scaffold00713:2265308..2265507]**

KDHFRSNKR**GMGPRPLRFG***

Short *flp-21* match supported by EST:

**>*Bx-flp-21*_[CJ985127]**

**MRTDALLKVAIFLFLAILAQLGQA**GPILESTNDLTALKMLRNYMERYGEPDGDVGYVVLDDMRSNKR**GMGPRPLRFG**

**>*Bx-flp-22*_[BUX_s01078.75]**

**MVAPMSLLFAFLLVVGHFLGQAQVVSG**SDIYQDNLYREAR**APAMKWMRFG**KR**SPQGKWMRFG**KR**APQGKWMRFG**KRADDVQDPEYE*

**>*Bx-flp-32*_[BUX_s00961.64]**

MPADKK**AMRNSLVRFG**KRDGAQAAPAAPVQPEFQMNYPVDSFGSHAQNPVFGYYRRSLHTEPRITPTLLQEFSADSDATTILRHTKKKPQHLLLQDTILFSTLLPSHA*

**>*Bx-flp-33*_[scaffold01198:748133..748332]**

KFFPNFY-AL-NFRRTVKTRR**APSGGDNYQWESMMQSLDNLRKPRFG***

**c.**

**>*Bx-nlp-2*_[BUX_s00351.431]**

**MRLLLALLFTLLAVGFA**DFLEPNELDQMEDIDVPRERR**SSLASGRWGLRPG**KR**SSLASGRWGLRPG**KRSIVDLVLEDDIEDESDKTRERR**NLASGRWGLRPG**KR**SLANGRWGLRPG**KRSMGMMPTQARHNPIMLLIPQL*

**>*Bx-nlp-3*_[BUX_s01281.317]**

**MSPFRGVSLASVLFAVLFLATTLSA**FLQDEVPLITEIEGVAKR**AINPFMDSIG**KRSGNVYVPGSRFYRHSLLFQRPSR**YFDSLAGQSLG**RR*

**>*Bx-nlp-7*_[BUX_s00579.689]**

***MMRSLILFLFGLLMPVLLA***KSEPSAGGVYEFNPRDWKADMSPHNFYRFRRYNVFSND**KRSEMEFDDPRYFSTAFGKR**ASSFL*

**>*Bx-nlp-8*_[BUX_s01141.33]**

MSRVPFLCRSYIILDSRVPKTGGALGEHKILGKVMRTIFTVLTVCFLQVLCLSQGQNTEDRSREQRYVLHKR**AFDRVDLSPFDFGAYS**KRYNNDLRSLYRKKK**SFDRLDQGPFGLV**KRKR**VFDRVDAGFGFG**KRSSGM*

**>*Bx-nlp-9*_[BUX_s00422.449]**

***MNLGVRTIDLFLWVSLFGILTQIVYGA***SFVQNLEDAELNEEDKR**GGGRAFLNTDD**KR**GGGRAFQFND**KR**GGARAFAPVDV**KR**GGARAFAPIDY**KR**GGGRAFSGFNGGNYYLLN**KR**GGGRSFYGGFNYGSLYAPSFPLSYE**KKADYVPYYLTEMKR**AGGRAFQPKYYDPYFWYVDQT**KR**AGGRSFPISDESKE**KRMLEEQADSRLAYAFRGPLA*

**>*Bx-nlp-10*_[BUX_s00422.89]**

***MRTVILCLILSVGCIGMASA***ASIYIDSNRPDYEENLQKVYDNRLQPYAIEYLAKR**AALPYSGGIYG**KR**AALPYSGGIYG**KR**ASVPFSGGIYG**KRSVDEAPVDHVNGISIR**AMPINGGFYG***

**>*Bx-nlp-12*_[scaffold00397:327542..327381]**

YRILTPEILQFGKRGDFRPLQFGKKDNYRPLQFGKRAAVVNHYLDLLPIDEIAN

Short *nlp-12* match supported by EST:

**>Bx-nlp-12_[CJ989213]**

***MKLYILLMLALVGWVCAAAA***SEEEADRFSRTDR**DYRPLQFG**KR**GDFRPLQFG**KK**DNYRPLQFG**KRAAVVNHYLDLL

**>*Bx-nlp-13*_[BUX_s01661.2]**

***MSTQTHFLAVVAATLCCLILCDA***EDAFYSIGDKR**NFDREFMHFG**KR**ATSEDFGRSFMPFG**KR**ATNDDFGRAFMPFG**KR**AANEEFGRAFMPFG**KR**ADAYDESFNRAFMPFG**KRSPMTLDQLIAKK**NMNRDFMHFG**KR**VAPSSFDREFMHFG**KRYV*

**>*Bx-nlp-14*_[BUX_s00252.98]**

**MTVHSRICLSAALFVMFCSAFVSS**AVVVRLPVAPTRSALFTKR**ALDALEGSDFGL**KRKR**ALDSLEGSDFGL**RKR**ALDSLEGSDFGL**RKR**ALDSLEGADFGL**KKR**ALDSLEGADFGL**KKR**ALDSLEGADFGL**RKR**ALDSLEGADFGL**RKR**ALDSLEGSDFGL**KKR**ALDALEGTGFGFD**KR**ALDMLEGSDFGL**KKR**ALDSLEGADFGL**KKR**ALDSLEGSDFGL**RKR**ALDSLEGSDFGL**KKRSPIARFYATGDDIRKLNDLKTQLEVELRRRLEKEAEA*

**>*Bx-nlp-15*_[BUX_s00508.98]**

MDRKR**SFDSFTGAGFTGMD**KR**SFDALTGSGFTGMD**KR**SFDSFTGAGFTGMD**RKRALARMVRDLKV*

**>*Bx-nlp-20*_[BUX_s00116.117]**

***MSPLLTTVTALFLFSSVYG***QRQNAITDK**VGQGNFAFA**RPFAFALTENGEWYPIAKR**SRTSTFAF**KETPKE**KE**R**DSFAFA**KR**SEDPKFAYDFA**KRAKFAFLKK*

**>*Bx-nlp-37*_[BUX_s00579.644]**

***MQSLVSSFVVLMTITVTVMTSKSDA***LPFPPHTSLVIQDTQLIRQFLDRYQPAVLFHPMVRRYDVQQEDMDDGEERLALRPMAKR**NNAEVVNHILKNFGTLDRLGDVG**K*

**>*Bx-nlp-38*_[BUX_s00294.125]**

***MVQKSAWQMLILLLLAVFSTIQA***DR**QWSAGVGLWG**KRSYPLLRKR**SPDFNPNGEAGLWDK**R**AQWQAANGLWG**KRSVPPVPQYVPWN*

**>*Bx-nlp-40*_[BUX_s00055.197]**

MNTAPAGEDLQKKIDAMEKHIQLLEKALLQRRSPLQGSLIEGNQIMNPRATRALAFQPMKR**MVAWQPM**KRSIAAEYNKDQVIRAIEEQLLEILHAGETLGVNAEEVLGDLKKKNGDLM*

**>*Bx-nlp-42*_[BUX_s00713.445]**

***MSLLTRILCTLTVVGITIAITHA***SPMDRSALKK**ASDNPQWEDLGWAWG**KRSVAVELVDPEDVMLQRYVRSMKAIKK**NPDWHDLGWAWG**K*

**>*Bx-nlp-46*_[scaffold01066:2702..2901]**

***MKNFVRKTKFKSMPLVFTNFFFIFQFDQTQS***SHPVAQKR**NIAIGRGDGFRPG**K

**>*Bx-nlp-47*_[scaffold01281:1738536..1738378]**

NLQSCLVLLLRLRVRPFFRQWKLRDHRRR**MLNYTDQWNRP**KRLFFRPKCVKSL
