# Supplementary figures and images for: Genomic Insights into the Origin of Parasitism in the Emerging Plant Pathogen Bursaphelenchus xylophilus
Source: PLoS Pathog. 2011 Sep 1;7(9):e1002219. doi: 10.1371/journal.ppat.1002219 (PMC3164644; doi:10.1371/journal.ppat.1002219)

Figure S1

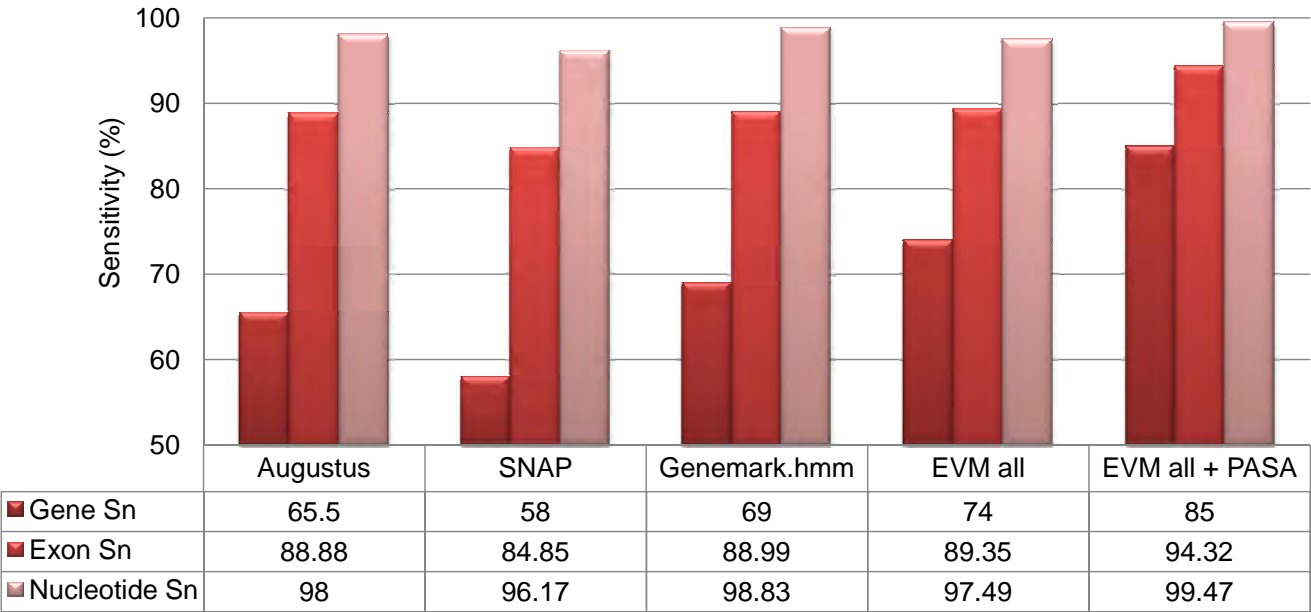

Supplement: Figure S1 — Accuracies of gene predictions by ab-initio methods and combined by EVidenceModeler (EVM). Gene prediction accuracy was calculated at the nucleotide, exon, and complete gene level using 200 manually curated gene models as references. Label Augustus, Snap and Genemark.hmm represent ab-initio gene predictions by each predictor. EVMall, which is the combined one by EVM with the optimised weight, includes the three ab-initio predictions and the GeneWise predictions based on pfam protein similarities. EVMall+PASA includes EVMall and Program to Assemble Spliced Alignments (PASA) alignment assemblies and corresponding terminal exon supplement. Sn, sensitivity. (PDF) [file ppat.1002219.s003.pdf]

Figure S2

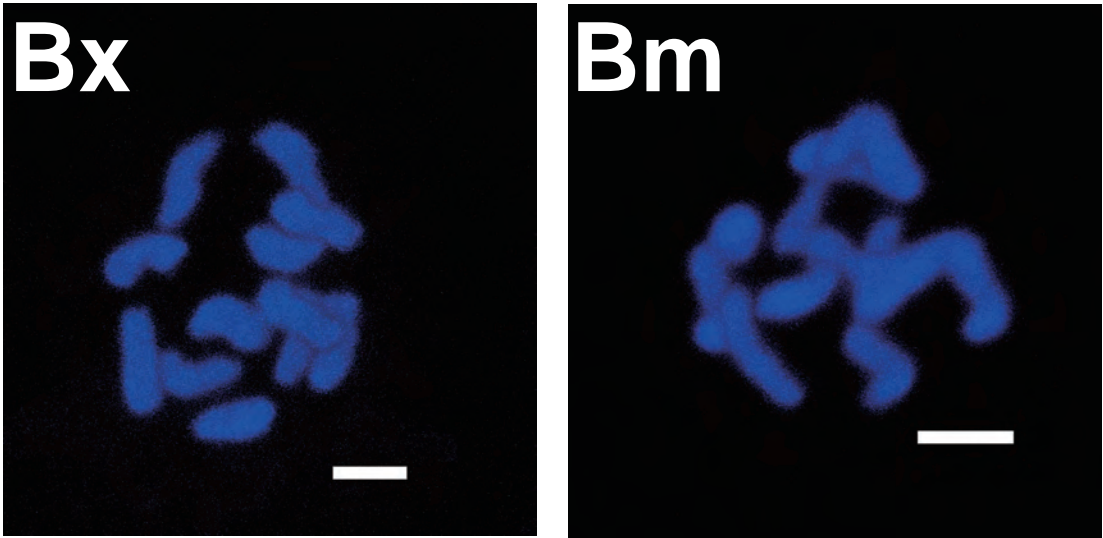

Supplement: Figure S2 — Chromosome numbers of B. xylophilus and B. mucronatus. B. xylophilus (Ka4C1 line) chromosome was observed to be 2n = 12. Bar = 2 µm. (PDF) [file ppat.1002219.s004.pdf]

Figure S3

C. elegans

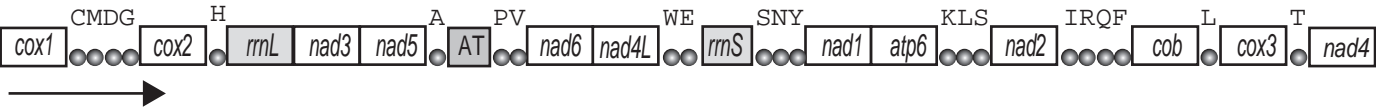

B. xylophilus\*

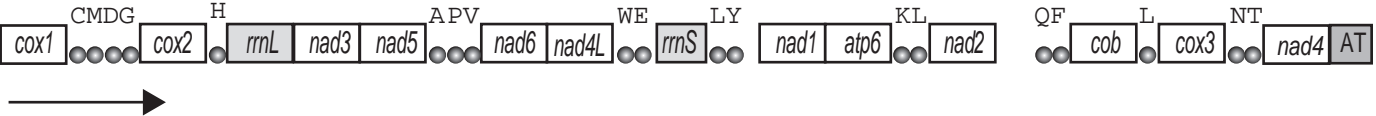

Supplement: Figure S3 — Gene orders of mitochondrial DNAs of B. xylophilus and C. elegans. Arrows indicate the direction of transcription of genes. The genomes contain 12 protein-coding genes (atp6, cob, cox1-3, nad1-6 and 4L), two rRNA genes (rrnS and rrnL), tRNA genes (circles with one letter codes to indicate transferred amino acid) and non-coding region (AT). Note that the B. xylophilus mtDNA sequence is not completed, lacking a short AT rich sequence. (PDF) [file ppat.1002219.s005.pdf]

Figure S4

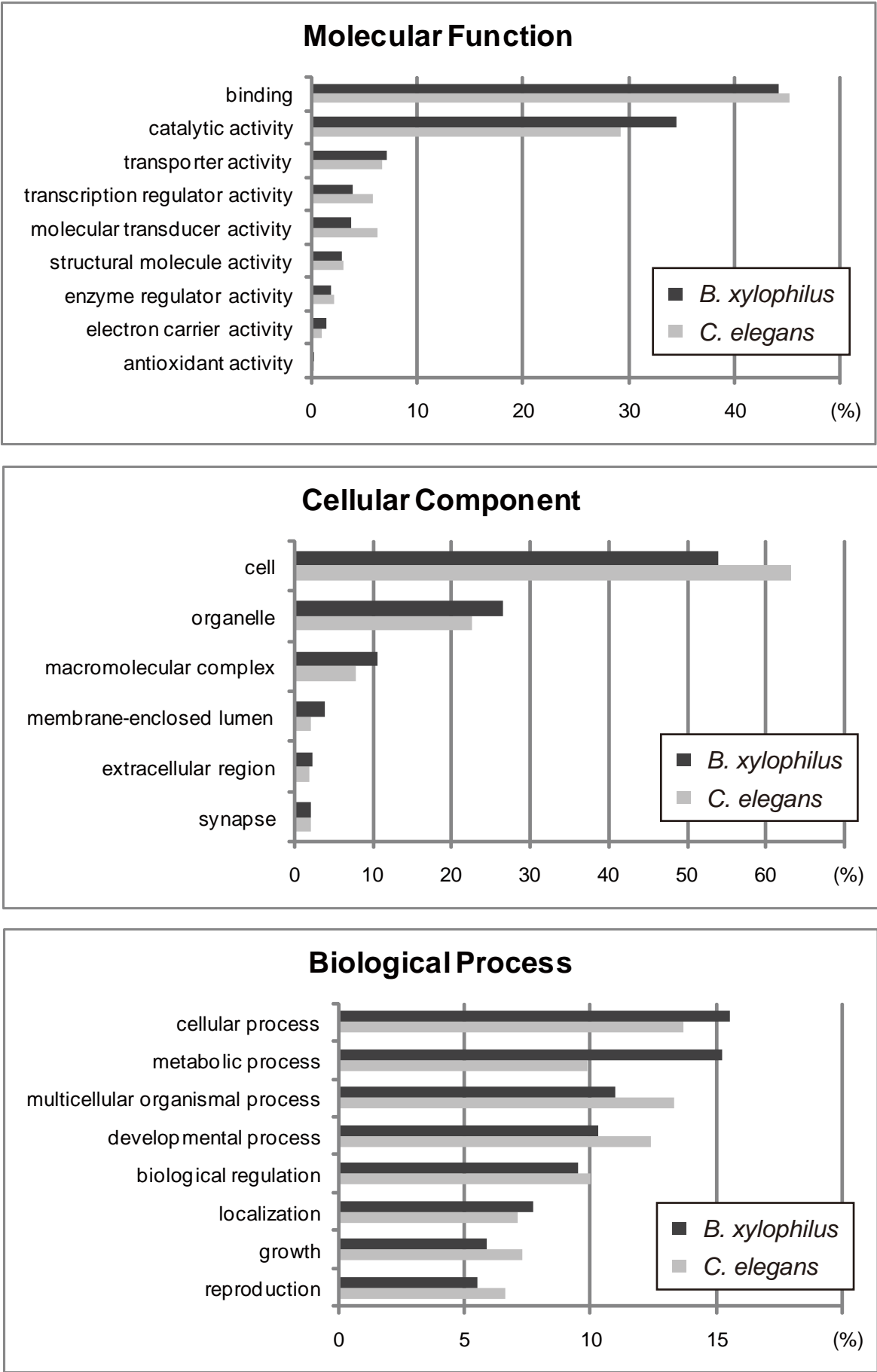

Supplement: Figure S4 — Distribution of the three major gene ontology categories assigned to B. xylophilus. Predicted genes can have more than one GO term. The x axis indicates the percentage of the term compared to the total of the terms. (PDF) [file ppat.1002219.s006.pdf]

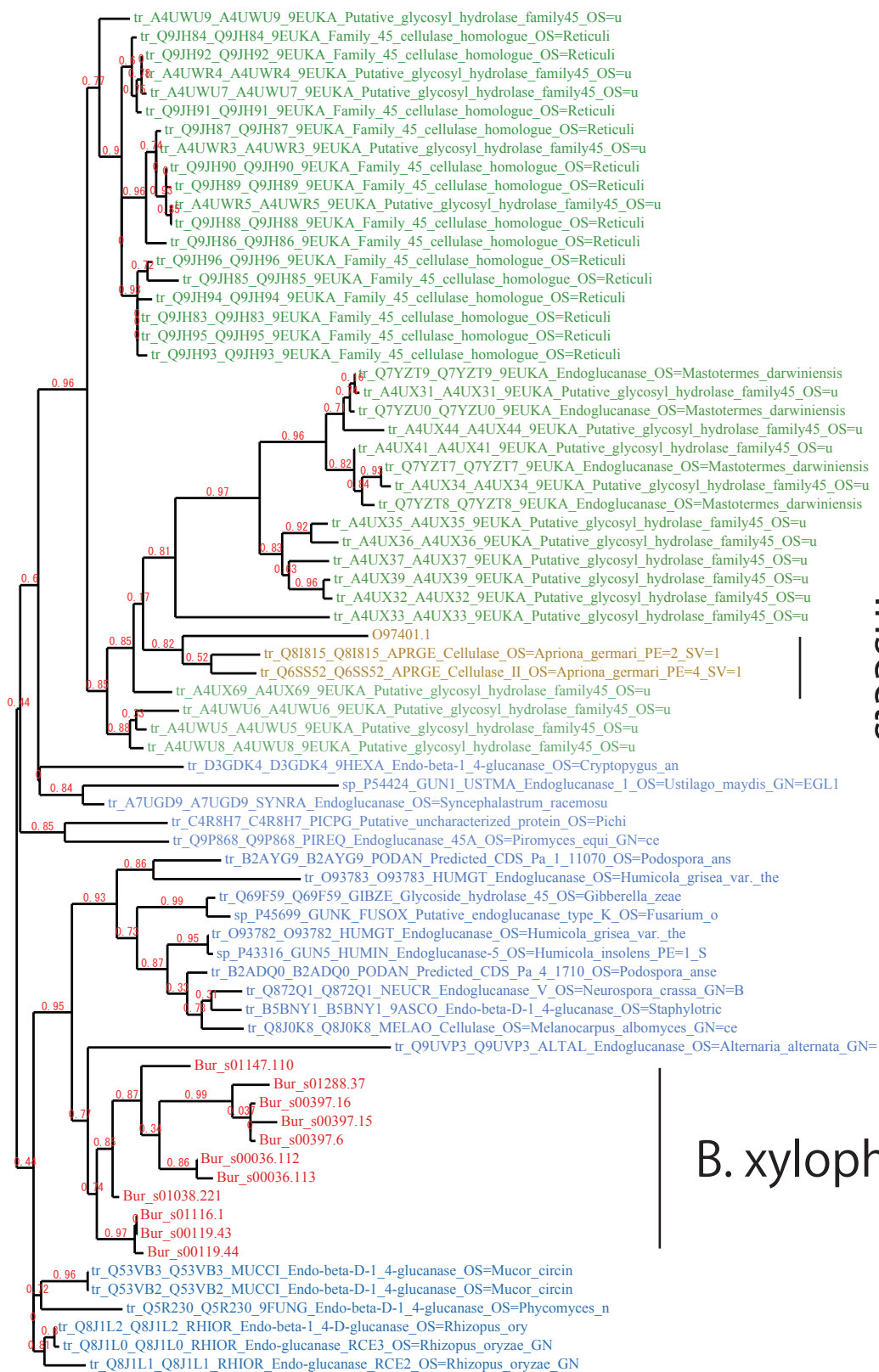

Protists

Insects

Fungi

B. xylophilus

Supplement: Figure S5 — Unrooted phylogenetic tree of GH45 proteins. Amino acid sequences of GH45 proteins in CAZy website (www.cazy.org) were retrieved from uniprot. The maximum likelihood phylogenetic tree was made using Phylogeny.fr (www.phylogeny.fr) using default setting. Proteins with short lengths or with long branches in the preliminary tree were removed from analysis. (PDF) [file ppat.1002219.s007.pdf]

Figure S6

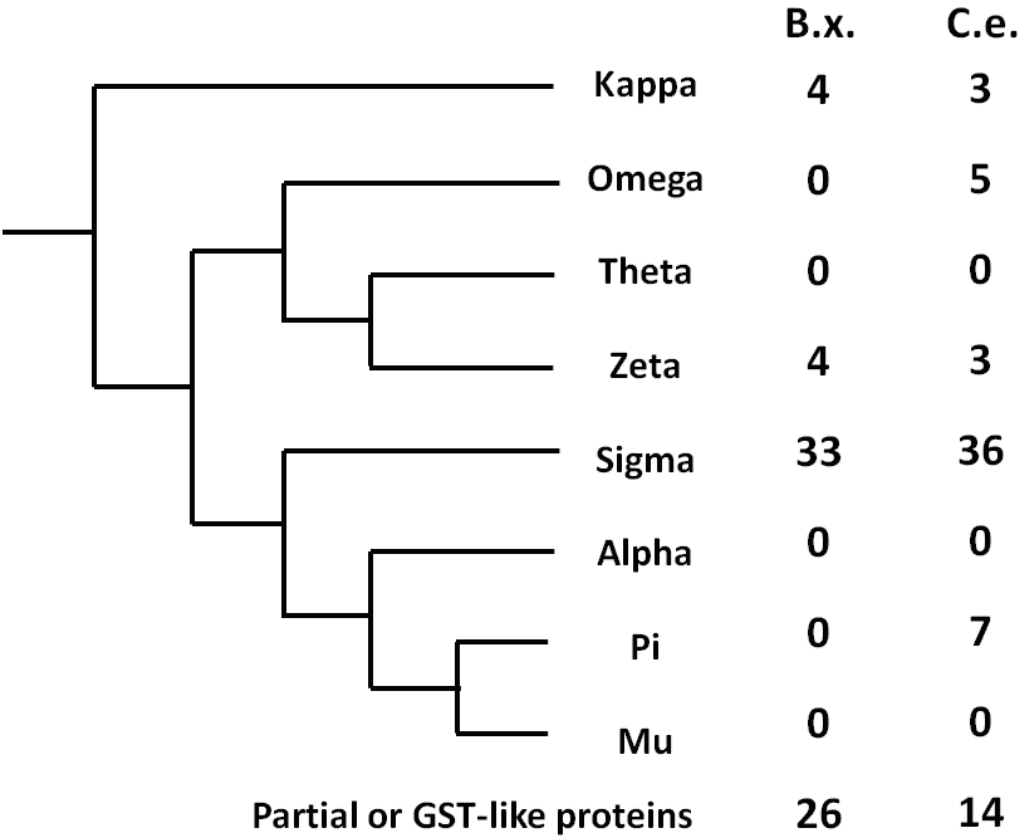

Supplement: Figure S6 — GST classifications in B. xylophilus and C. elegans. Full-length GSTs containing both conserved N and C domains are classified into the classes. GST phylogeny tree is reconstructed according to Zimniak & Singh [91]. (PDF) [file ppat.1002219.s008.pdf]

Figure S7

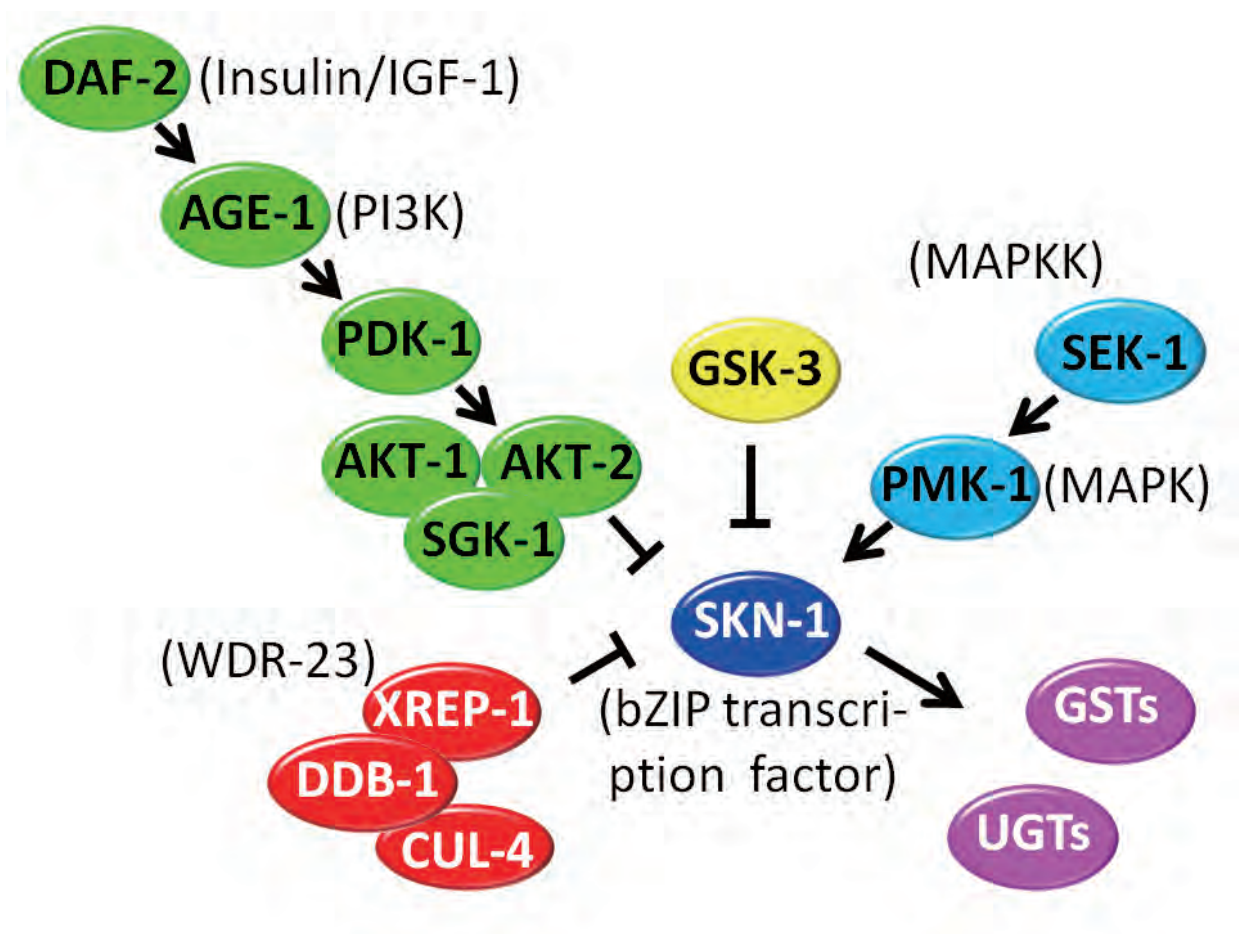

Supplement: Figure S7 — Signalling pathways involved in phase II enzyme expression. The XREP-1/DDB-1/CUL-4 complex (red), the DAF-2 pathway (green), and glycogen synthase kinase (yellow) negatively regulate SKN-1 (dark blue), whereas the p38 MAPK pathway (light blue) positively regulates SKN-1. The relationships among these pathways are not clear. When animals are exposed to xenobiotics, SKN-1 accumulates in the nucleus and induces the expression of many phase II enzymes. C. elegans protein names are followed by more general names in brackets. (PDF) [file ppat.1002219.s009.pdf]

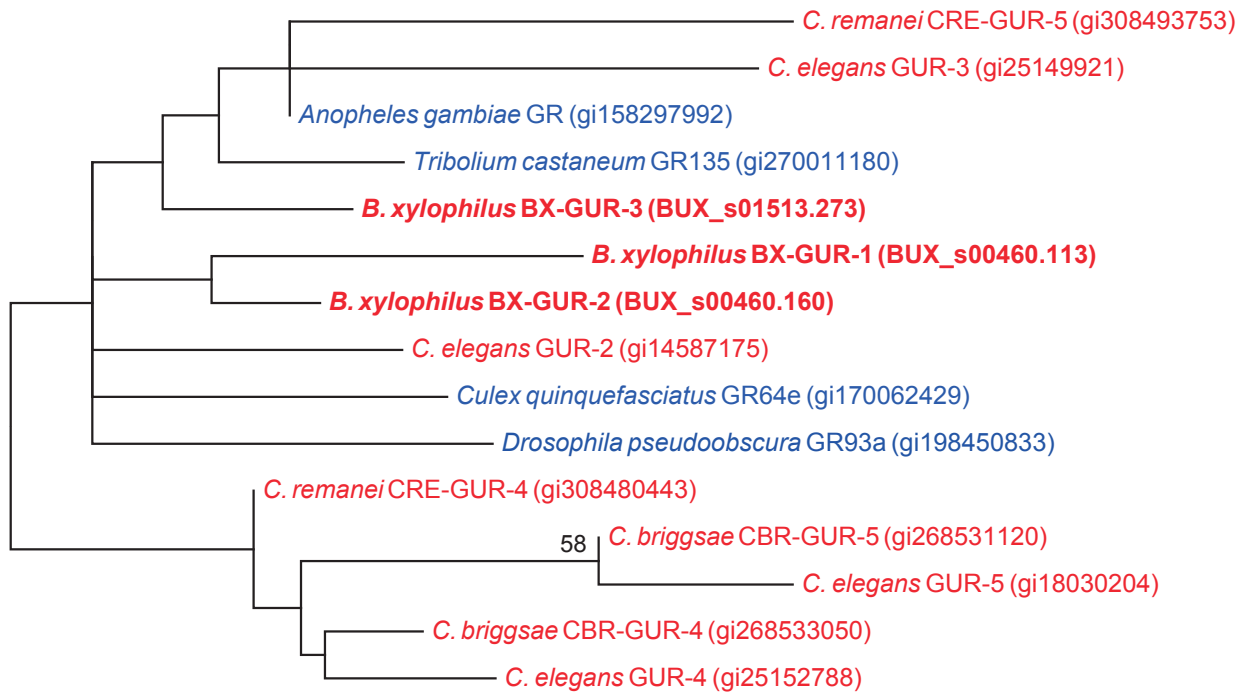

1

Supplement: Figure S8 — Phylogenetic tree of 16 gustatory receptors. The phylogenetic tree was built by maximum likelihood method according to MEGA5 based on the JTT matrix-based model with uniform rate. Three proteins of B. xylophilus, (GUR-1, -2 and -3 indicated with bold) proteins of nematodes C. elegans, C. remanei, C. briggsae (GUR indicated with red), and proteins of insects Drosophila pseudoobscura, Anopheles gambiae, Culex quinquefasciatus, Tribolium castaneum (GR with blu) collected from NCBI protein database were used in the analysis. The scale bar indicates number of amino acid changes per site. Bootstrap values more than 50% form 1000 replications were shown on appropriate branches. (PDF) [file ppat.1002219.s010.pdf]

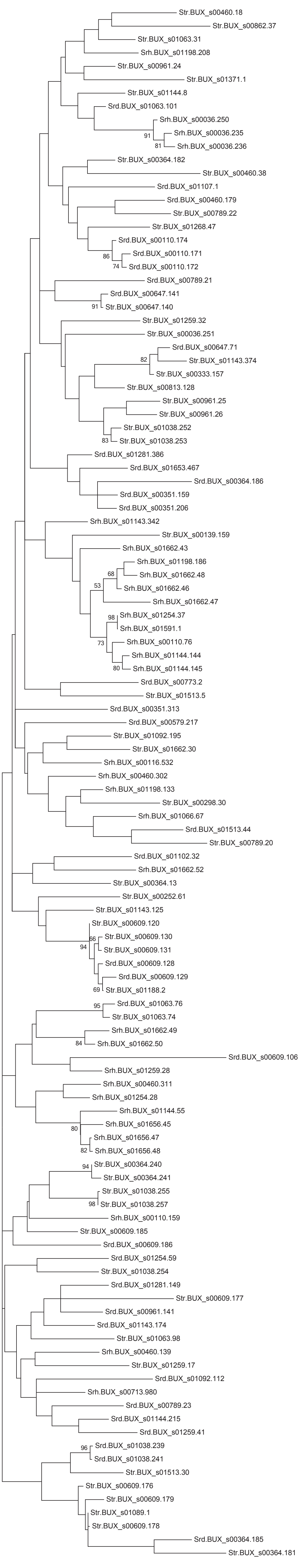

1

Supplement: Figure S9 — Maximum likelihood tree of STR superfamily chemoreceptor proteins in B. xylophilus. The phylogenetic tree was built by maximum likelihood using MEGA5. All 5 families (Str, Srd, Srh, Sri and Srj) from Str superfamilies of B. xylophilus were included in the tree. The scale bar indicates number of amino acid changes per site. Bootstrap values more than 50% were shown in the tree. (PDF) [file ppat.1002219.s011.pdf]

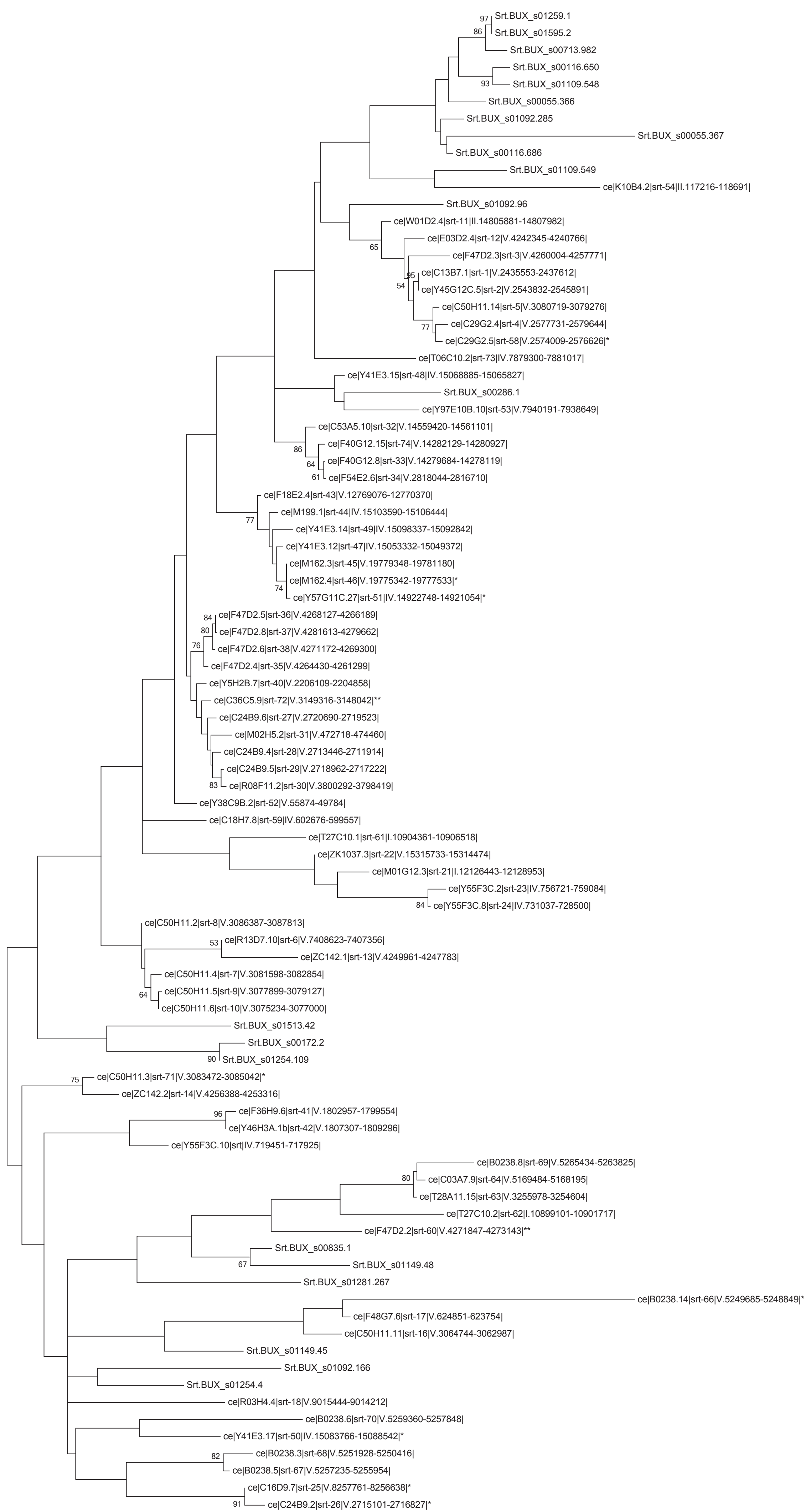

Supplement: Figure S10 — Maximum likelihood tree of SRT family chemoreceptor proteins in B. xylophilus and C. elegans. The phylogenetic tree was built using MEGA5. The scale bar indicates number of amino acid changes per site. Bootstrap values more than 50% were shown in the tree. (PDF) [file ppat.1002219.s012.pdf]
