# Supplementary material for: Genomic Insights into the Origin of Parasitism in the Emerging Plant Pathogen Bursaphelenchus xylophilus
Source: PLoS Pathog. 2011 Sep 1;7(9):e1002219. doi: 10.1371/journal.ppat.1002219 (PMC3164644; doi:10.1371/journal.ppat.1002219)
Supplement: Text S1 — Supplementary text – Detailed Result. (DOC) [file ppat.1002219.s013.doc]

Genomic insights into the origin of parasitism in the emerging plant pathogen *Bursaphelenchus xylophilus*

Kikuchi et al.

Supplementary Text – Detailed Results

**Sequencing and Assembly**

The chromosome number of the Ka4C1 inbred line of *B. xylophilus* was determined to be 2n=12 (Figure S2) by microscopic observations. In addition, the chromosome number of a closely related species, *Bursaphelenchus mucronatus,* was also found to be 2n=12. The genome size of *B. mucronatus* has previously been estimated to be about 30 Mb by flow cytometry [1]. Using a real-time PCR method the size of the *B. xylophilus* genome was estimated at 69.0 ± 5.5 Mb (Table S1 in Text S2).

Genomic DNA was extracted from Ka4C1 inbred line nematodes and used to generate llumina GAI reads and 454 FLX reads including single, 8k mate-pair and 20k mate-pair reads. A combined assembly strategy assembled these reads into 1,231 scaffolds (see Materials and Methods). The calculated genome size of the assembly was about ~74.5 Mb with a contig N50 value of 18.1kb and a scaffold N50 value of 1.16 Mb (Table 1).

**Mitochondrial genome**

A scaffold (scaffold00976), 13,410 bp in length, showed significant BLAST similarity to known nematode mitochondrial genomes. 12 mitochondrial proteins, 2 rRNAs were predicted via similarity search and 19 putative tRNAs were predicted by tRNAscanSE on the scaffold. The scaffold was likely to contain most of *B. xylophilus* mitochondrial sequence, but seems to lack a short AT rich sequence, possibly because this region lacks the complexity required for proper assembly. The arrangement of the genes within the mitochondrial sequence was similar to that of *C. elegans* (Figure S3).

**Repeat analysis**

A total of 201 different transposable element related sequences were found through homology searches to known TE elements and *de novo* repeat programs (see Materials and Methods). The long terminal repeat (LTR) retrotransposons are the most abundant group, occupying approximately 0.8% of the genome. The observed repeat content is likely to be an underestimate as some of these consensus sequences were found spanning the gaps in the scaffolds and in smaller contigs. The longest transposable element was a LTR retrotransposon 7.7kb in length with significant matches to Pao retrotransposon peptidase present both in the Nematoda and Apicomplexa. However, only one full copy and 10 shortened fragments of this retrotransposon were present in the assembly. This suggests that the TE is ancient and that activity is present but low.

**Noncoding RNAs**

A set of 729 genes with acceptors for all 20 amino acids was identified by tRNAScanSE [2]. This set was composed of 415 true genes, 300 pseudo genes and 14 putative tRNAs with unknown isotypes (Table S2 in Text S2). These hits were distributed across 241 scaffolds in the assembly and 27 of them contained putative introns.

The genes for three of the four ribosomal RNA (rRNA) components, 18S, 5.8S, and 26S, are known to occur in large, tandem-repeat structures in *C. elegans* and other higher eukaryotes [3]. There are thought to be 100–150 copies of this repeat in *C. elegans*. We found 17 supercontigs (5 scaffolds and 12 orphan contigs) containing sequences that showed similarity to *C. elegans* rRNA (genbank no X03680) and/or *B. xylophilus* rRNA fragments available in Genbank at E-value <1e-10. However no contig contained a complete 18S-5.8S-26S unit. This may be because assembly of repetitive regions can be problematic, as seen in genome assemblies in other organisms [4]. When short read sequences (Illumina GAI reads) were mapped to the assembly, the rRNA regions showed higher coverage (~100 times) than the average of other regions (data not shown), suggesting high copy numbers of rRNA genes present in the genome.

Trans-splicing is a process found in a variety of organisms including many nematodes. It involves the addition of a small leader RNA sequence, known as the spliced leader (SL), to the 5’ end of many pre-mRNAs. In nematodes, two types of *trans*-splicing have been described. The most conserved form is spliced leader 1 (SL1) a 22-nt sequence *trans-*spliced to the 5’ end of many pre-mRNAs [5]. In *C. elegans* a distinct SL sequence called SL2 is *trans* spliced to the 5’ end of genes present as operons. However, in other nematodes, including *B. malayi* and *M. incognita*, SL2 trans-splicing appears not to exist [5]. We identified 25 SL1-like sequences in the assembly of *B. xylophilus* but no SL2-like sequence was identified. The SL1-like sequences were distributed in 16 supercontigs (6 scaffolds and 10 orphan contigs) and all of them were close to 5S RNAs. This is similar to *C. elegans* whose SL1 sequences exist in the same tandem repeat fragments as 5S rRNAs.

**Automatic annotation (GO, InterPro, KOBAS)**

As an initial functional characterization of the *B. xylophilus* proteome, we used HMM searches against the Pfam24 database to find known domains and functional sites. The top ten matches with an E-value less than 1e-5 were recovered. For comparison, similar analysis was performed using the *C. elegans* proteome (wormpep211). There were 12,134 matches in *B. xylophilus* and 20,666 matches in *C. elegans*. We found a notable increase in *B. xylophilus* for Pfam hits related to protein lysis including Eukaryotic aspartyl protease and Peptidase C1A (see section Peptidases and lysosome activity for more details).

A GO term was assigned to each of the *B. xylophilus* proteins. For the GO term assignment we used a BLASTP search against NCBI non-redundant proteins as well as InterProScan. From a total of 18,074 proteins, 12,426 (68.8%) showed hits to proteins present in the nr database (cutoff 1e-10) and 9,079 (50.2%) were assigned GO terms (Figure S4).

*B. xylophilus* proteins were then mapped to the KEGG pathway and pathways significantly increased or decreased in *B. xylophilus* compared to *C. elegans* were identified using KOBAS software [6] (Table S3 in Text S2). Proteins frequently mapped on the lysosome pathway included a variety of families of cathepsins (peptidases) (for more details, see sections on Peptidases and Utilisation of plant secondary metabolites).

**OrthoMCL**

Out of a total of 231,879 predicted proteins from the 10 rhabditid nematode genomes included, the OrthoMCL pipeline removed a total of 91 that were either very short (less than 10 aa) or of dubious quality (>20% stop codons within the predicted reading frame). The remaining 231,788 were classified into 27,547 families of orthologues, and an additional 51,942 singleton proteins. Families ranged in size from 2 (the modal size) to 817 genes, with a median of 5. We base our phylogenetic inference (Figure 5) on a total of 19,356 aligned nucleotide positions from 23 gene families present as a single copy in each genome and for which amino-acid alignments contained less than one gap for every 10 positions, out of a total of 253 universal single-copy families. If we assume that nucleotide substitutions are occurring at an approximately equal rate, on average, our molecular phylogeny confirms the ancient divergence between the Aphelenchoidea (*B. xylophilus*) and Tylenchida (*Meloidogyne* spp.), which contain the plant parasitic nematodes for which genomic data is available [7]. Indeed, *Bursaphelenchus* is only very distantly related to any other nematode for which genome sequences are available.

Figure 5 shows the distribution and evolutionary dynamics (birth and loss) of these gene families across the 10 genomes. In the long period of time for which *B. xylophilus* has diverged from its nearest relatives for which genomic data is available, there has been relatively little change in gene family composition (a total of 1380 gains and losses), perhaps reflecting the relative lack of ecological specialization of *B. xylophilus* compared to the sedentary plant parasites, which show far more dynamic gene family content over the same time period (totals of 3751/4932 events in two *Meloidogyne* spp.) and particularly lose a much greater number and range of gene families compared to *Bursaphelenchus*. Indeed, as much gene family diversity is seen between pairs of species within the genus *Caenorhabditis*, over a far shorter time period. In this respect, *B. xylophilus* may represent the closest model of an ‘ancestral’ nematode genome of any of the published genomes to date, including that of the specialised intestinal parasite *Trichinella spiralis* [8], which forms an outgroup to the Rhabditida genomes analysed here, but that has lost many gene families and gained a number of lineage-specific families.

Figure 6 illustrates a number of patterns of gene sharing that could have biological meaning. In general, the numbers of gene families shared between different species does not appear to strongly reflect the phylogenetic relationships between species – for example, *Brugia*, the most divergent genome of the 5, has only 1,369 unique families, and *Meloidogyne* and *Bursaphelenchus*, share very few genes. These results show that gene family content is highly labile between different nematode species, supporting the picture of extensive genetic diversity of nematodes [9]. Gene families shared by ecologically similar species, are candidates for genes most important in these functions, although the small number of comparisons available and the fact that ecology and evolutionary history are conflated in several of these comparisons make these inferences rather uncertain with the existing sample of nematode genomes.

**Peptidases and lysosome activity**

The MEROPS database [10] classifies peptidases into six catalytic types, i.e. aspartic, glutamic, cysteine, metallo, serine and threonine peptidases. Proteins belonging to families A01A, C01A, C13, M01 and M13 are particularly expanded in *B. xylophilus* (Table S4 in Text S2). A01A proteins are acidic peptidases and their function is thought to be the external digestion of food proteins by saprophytic organisms. Proteins in the M13 family are thought to act outside animal cells to degrade or convert polypeptide substrates. C01A families contain many cathepsins, which contribute proteolytic activity to the lysosomal system of eukaryotic cells. Proteins in the C13 family are Legmains which are most active at acidic pH and have been shown to work in the lysosome.

**HGT gene candidates**

To look for potential horizontal gene transfers into the *B. xylophilus* lineage, we surveyed all *B. xylophilus* proteins. Out of a total of 223 candidate HGT genes from our BLAST-based analysis, we conservatively accepted a total of 24 as being phylogenetically well-supported. Other HGT candidates showed ambiguous phylogenetic signals, for example where nematode sequences clustered with some other animal sequences, or where there was no clear monophyly of major phylogenetic groups, suggesting that there may be little phylogenetic signal for these genes, or that extensive HGT has complicated the evolutionary history of these genes. Our inference of HGT is deliberately conservative, so additional, more detailed phylogenetic work is likely to reveal additional examples of laterally transferred genes.

Table 6 lists the HGT genes we have identified. The two families of cell-wall degrading enzymes and one peptidase family are discussed in the main text. Our HGT candidates include at least one other carbohydrate-degrading enzyme, homologous to a bacterial 6-phosphogluconolactonase, and a HAD-superfamily hydrolase that could act on any of a wide range of possible substrates.

The HGT genes we identify here come from a range of different bacterial and fungal sources, but all are at least potentially present in the environment of *B. xylophilus*. It is tempting to speculate that these other laterally transferred enzymes are involved in parasitism, although it is, of course, equally possible that the *Bursaphelenchus* genome acquired these genes for other functions, even if it were via the same mechanism as the cell-wall degrading enzymes were recruited.

**Effector candidates**

We examined the predicted protein set from *B. xylophilus* for homologues of cyst and root knot nematode effectors, other than cell wall modifying proteins. Some significant matches to effectors from all three species were found (Table 7). However, the *B. xylophilus* sequences that matched these effectors either did not have predicted signal peptides or, if a signal peptide was predicted, homologues were also present in a wide range of other species including *C. elegans* and animal parasitic nematodes. Both these lines of evidence suggest that the *B. xylophilus* homologues identified in this analysis are not true effectors that play a role in parasitism.

A previously described bioinformatics approach [11] was subsequently used to seek novel candidate effectors from *B. xylophilus*. Using this analysis, 2,297 of the *B. xylophilus* predicted proteins were identified as being potentially secreted as they had a signal peptide but lacked a putative transmembrane domain. BLAST searching this dataset with previously characterized *B. xylophilus* cell wall modifying enzymes (cellulases, 1,3 endoglucanases, pectate lyases and expansins) confirmed that all were present, providing reassurance as to the validity of the approach. 923 of the predicted secreted proteins represented novel sequences, producing no significant matches against the NR database (Dataset S1). Even allowing for the false positives that will inevitably be produced using a bioinformatic approach these sequences seem likely to contain novel proteins that may be involved in host-parasite interactions.

**Utilisation of host secondary metabolites**

The response to stress conditions is important for all organisms. SKN-1, a *C. elegans* Cap’n’collar (Cnc) transcription factor regulates expression of many detoxification enzymes [12]. In the absence of oxidative stress or electrophilic xenobiotics, SKN-1 seems to be degraded through the ubiquitin-proteasome machinery of the XREP-1(or WDR-23), DDB-1, and CUL-4 complex. However, under stress conditions SKN-1 escapes from the complex, accumulates in the nucleus and induces the expression of its target genes [13,14,15]. SKN-1 is also controlled negatively by GSK-3 [16] and the Insulin/IGF pathway [17], and positively by the p38 MAPK pathway [18]. Orthologues that may be involved in SKN-1-controlled pathways (shown in Figure S7) were all identified in *B. xylophilus*.

Activated SKN-1 induces the expression of many (but not all) phase II detoxification enzymes, which include glutathione S-transferase (GST) and UDP-glucuronosyl transferase (UGT) [12,14,15,19,20]. GSTs and UGTs execute a central role in protecting organisms against a variety of xenobiotics. We identified 41 full-length (containing both N and C domains) GSTs, 26 partial GSTs or GST-like proteins, and 60 UGTs in the *B. xylophilus* genome. Full-length GSTs were classified into 4 Zeta, 4 Kappa, and 33 Sigma classes (Figure S6). High redundancy of sigma class GSTs is a similarly observed trait in *C. elegans*.

Phase I enzymes of cytochrome P450s (CYPs) are also important for xenobiotic metabolisms. Seventy-six full-length and partial CYPs were detected in the *B. xylophilus* genome; among them, seven belong to CYP4 family, two CYP13, one CYP29, two CYP32, 53 CYP33, while the remaining 11 are partial or unknown CYPs. WormBase (release211) lists 81 CYPs, a number similar to the 76 CYPs found presently in *B. xylophilus* (Table 8).

ATP-binding cassette (ABC) transporters, sometimes called Phase III enzymes, utilize ATP to actively pump out detoxified xenobiotics. One hundred and six ABC transporters were detected in the *B. xylophilus* genome; this number was about twice that for *C. elegans* and about three times that for *M. incognita* (Table 8).

*B. xylophilus* has a large number of detoxification enzymes as do the free-living soil nematode *C. elegans* and the necromenic nematode *P. pacificus* [21], which may reflect the variety of stressful environments that it encounters in pine trees and in insects during its life cycle.

**Development (Embryogenesis and Dauer Formation)**

We identified orthologues of the majority of *C. elegans* proteins involved in embryogenesis in *B. xylophilus,* including sperm-derived CDK-4, cytoskeleton-component proteins and all of the cortical proteins except for PAR-2. The set of *C. elegans* cortical proteins PAR-3/PAR-6/PKC-3 (sometimes called the PAR complex) is known to be well conserved throughout metazoans [22] and it is therefore not surprising that this complex from *B. xylophilus* is highly similar to that in *C. elegans*. Neither the PAR-2 downstream target LET-99 or GPR-1 and -2, which are important for the first cell division of the *C. elegans* embryo [22] were obvious in the *B. xylophilus* genome. Although a putative homologue of PAR-1, a serine/threonine kinase protein, was present in *B. xylophilus*, it was considerably smaller (467 AA) than that in *C. elegans* (1,192 AA) and may not function in unequal cell division. Unique PAR-1 distributions in two rhabditid nematode embryos (*Diploscapter* sp. and *Protorhabditis* sp.) were reported recently [23]. Although the first cell divisions in these two nematodes are asymmetric, PAR-1 distributes itself all around the cortex. In contrast to the broadly conserved PAR complex (PAR-3/PAR-6/PKC-3), the absence or presence of PAR-1 and its relatives (PAR-2 and other unknown proteins) may be species specific and could generate diversity.

A great deal is known about the genetics underlying dauer formation in *C. elegans.* Multiple pathways involving insulin-related/IGF growth factor, transforming growth factor (TGF)-β and guanylyl cyclase regulate *C. elegans* dauer larva formation and recovery [24]. Parasitic nematodes also have dauer-like (often infective) stages but less is known about the molecular mechanisms controlling this process in these organisms. Recently the Daf12-Daf-9-related pathway, which functions downstream of the former three pathways and is required for dauer development in *C. elegans*, was shown to be conserved in other nematodes including *P. pacificus* and *Strongyloides papillosus* [25]. In contrast, studies on the orthologue of *daf-7* in parasitic nematodes indicate substantial functional divergence during infective larvae formation (reviewed in [26]).

In *B. xylophilus,* orthologues of most genes involved in these pathways were identified (Table S5 in Text S2). We also identified orthologues of three clustered DAF-22 genes, three dispersed DHS-28 genes and four ACOX-1 genes which are involved in *C. elegans* dauer pheromone synthesis in the *B. xylophilus* genome [27,28]. *B. xylophilus* enters the third-stage dauer larva (DL3 or dispersal third-stage larva LIII) under adverse conditions (Figure 2). Like *C. elegans*, this DL3 formation is influenced by environmental conditions such as food, temperature and/or nematode density [29]. Pathways that respond to these environmental cues, which are shared by *C. elegans,* may be more conserved in *B. xylophilus* than in other parasitic nematodes, most of which use different cues when forming a dauer stage.

In addition to DL3, *B. xylophilus* has a specialized stage called the fourth-stage dispersal larva (DL4 or LIV), which morphologically and physiologically corresponds to *C. elegans* dauers, *e.g*., degeneration of the digestive system occurs. *B. xylophilus* DL3, develop into the DL4 when stimulated by the presence of the vector beetle *Monochamus alternatus* and become ready to board the vector [29,30]. Previous studies showed that several novel genes are expressed specifically in the DL4 nematodes [31]. These observations suggest that *B. xylophilus* uses different type of environmental stimuli to control this part of the lifecycle and different pathways and proteins may be involved in this part of the life cycle.

**Neuropeptide-encoding genes**

In the phylum Nematoda at least 47 *nlp* genes, 33 *flp* genes (29 in *C. elegans*, 4 in various other species) and 40 *ins* genes (incorporating 39 *ins* genes and *daf-28*), encoding >270 distinct peptides have been described (for review see [32]). These peptides have diverse effects on nematode biology: FLPs impact neuronal/neuromuscular transmission, sensory activities and control some behaviours; NLPs effects are diverse and include the modulation of behaviour, sensory perception, growth and development; INSs regulate organismal growth, development and reproduction in response to external stimuli. The *B. xylophilus* genome contains 21 *flp*, 17 *nlp* and 7 *ins* gene orthologues (Table S6 in Text S2; Dataset S2), numbers which, while falling short of *C. elegans*, compare favourably with the closest available comparator, *M. incognita*, for which 19 *flp* genes and 22 *nlp* genes have been reported [33].

**Chemoreception**

In *B. xylophilus* three classes of potential chemoreceptors were considered: a) gustatory receptors, b) serpentine receptors, c) ionotropic glutamate receptors and d) others.

1. Three *B. xylophilus* genes display significant similarity to gustatory receptor genes in nematodes. Based on similarity and transmembrane structure, these receptors could represent an unusual chemosensory gene family with a common origin with insect gustatory receptors that has remained small in nematodes but which has exhibited substantial lineage expansion in insects. Phylogenetic analyses revealed that gustatory receptor genes usually displayed high divergence between individual gene. In comparisons within insects, between insects and nematodes, and within nematodes, most individual nematode receptors occupied widely different branches of the phylogenetic trees regardless of which method was used (Figure S8). Gustatory receptors of nematodes and insects were predicted to have the intracellular amino-termini, whereas classical GPCRs have the inverted topology [34].
2. The main group of putative chemosensory genes in nematodes is represented by GPCRs. InterProScan and BLAST comparisons with canonical *C. elegans* receptors revealed a substantial number of serpentine receptors *in B. xylophilus*, with similarity to most of the gene families identified in *C. elegans* [35]. However, phylogenies of *B. xylophilus* serpentine receptors could not be readily adapted to the families described in *C. elegans* (Figure S9). Comparisons between receptors in the two species indicate that many receptors in *B. xylophilus* represent lineage-specific expansions and duplication (Figure S10). We find 118 genes from *Str* superfamily, 26 from *Srg*, 6 from *Sra* and 26 from two other families.
3. A third potential forest of chemosensory genes is represented by the ionotropic glutamate receptor family. A substantial fraction of this family has become adapted for a chemosensory function in *D. melanogaster* (Dmel Ir) [36]. Interproscan and BLAST comparisons of individual Dmel IRs with chemosensory function revealed several orthologues in *B. xylophilus* (7 genes). However, all of these genes also displayed extensive cross-homology with other ionotropic glutamate receptors in many different organisms, which obscures any direct link to insect chemosensory IRs. Several families of GPCRs that may be receptors for neurotransmitters including bioorganic amines (dopamine, serotonin/octopamine, tyramine), gamma- aminobutyric acids, acetlycholine and neuropeptides were present and may be used for chemoreception.
4. Genes encoding several components of chemosensory signal transduction pathways were found including G proteins (21 G-alpha subunits and 2 regulators of G protein signalling) and 2 GPCR kinases. Apart from GPCRs, transient receptor potential channels, possibly for chemosensory genes, were characterized in *B. xylophilus*. Intracellar binding proteins (lipocalin/cytosolic fatty-acid binding protein) were found in *B. xylophilus*, but there were no extracellar binding proteins such as those that exhibit lineage expansion in insects. *B. xylophilus* homologue of chemosensory mutant for *odr* (odorant response abnormal)-2 in *C. elegans* was identified.

**RNAi pathway**

RNAi pathway effectors comprise five main functional groupings which were used here to define RNAi pathway-associated genes in *B. xylophilus*. These are small RNA biosynthesis, double stranded (ds)RNA uptake, amplification and spreading, Argonautes [AGOs] and RNA-induced silencing complex [RISC]-associated, RNAi inhibitors and nuclear effectors. The 37 effector homologues the *B. xylophilus* genome encodes is more than found in the root knot nematodes *M. incognita* (27) and *M. hapla* (28) (unpublished data; [33,37]).

The *B. xylophilus* genome encodes putative orthologues for eight of the nine small RNA biosynthetic protein-encoding genes considered in our dataset (Table S7 in Text S2). In contrast, orthologues of the dsRNA uptake and spreading proteins are not well represented in *B. xylophilus*, as with other parasitic nematodes (unpublished data). No orthologues of *sid* genes were identified, with *rsd-3* being the only representative dsRNA uptake / spreading gene in *B. xylophilus*. RNA-dependent RNA polymerase (RdRp) protein encoding genes are expanded relative to *C. elegans*, with four ego-1-like orthologues, two *rrf-1*-like, and three *rrf-3*-like RdRp’s (Table S8 in Text S2).

Sixteen putative Argonaute (AGO) genes were identified relative to the 27 of *C. elegans*. These included the highly conserved miRNA-interacting AGOs (*alg-1* and *alg-2*), the exogenous RNAi effector *rde-1* which is poorly conserved across other plant and animal parasitic nematodes (unpublished data), and an expanded family of six discrete R06C7.1-like AGOs, two CSR-1-like AGOs, and two ZK1248.7-like AGOs, among others. Further analyses of functional catalytic and RNA-binding MID subdomains (within the PIWI domain) indicate a degree of divergence in some of the predicted AGOs from the *C. elegans* orthologue (*alg-2*, *rde-1*, r06c7.1b, r06c7.1c, r06c7.1e, r06c7.1f, t23d8.7, y49f6a.1, zk1248.7a and zk1248.7b [10 of the predicted 16]). Genes encoding other RISC cofactors were identified, including *ain-1*, *tsn-1* and *vig-1* (Table S9 in Text S2).

Surprisingly, the short interfering RNA (siRNA) inhibitor *eri-1*, which is highly conserved in other nematode spp. (unpublished data), was not found in the *B. xylophilus* genome. MicroRNA (miRNA) inhibitors, such as *somi-1* and two *xrn-2*-gene orthologues were identified (Table S10 in Text S2).

The RNAi pathway also includes a series of nuclear effectors, involved in processes such as methylation and chromatin modification. These genes were reasonably well represented in the genome of *B. xylophilus*, relative to other nematode spp. outside the Caenorhabditis (Table S11 in Text S2).

**Analysis of synteny with *C. elegans***

We wanted to examine the extent of chromosomal rearrangement between *B. xylophilus* and *C. elegans*. It has been shown that *C. elegans* chromosomes display macrosyntenic relationship with those of the more distantly related *Trichinella spiralis* [8]. That is to say that a particular region of the *T. spiralis* genome contains genes orthologous to those from predominantly one *C. elegans* chromosome, but that these are interspersed by genes orthologues to those from other *C. elegans* chromosomes. We used OrthoMCL to determine orthologous groups with a single gene in both *B. xylophilus* and *C. elegans* (4009 genes). We painted the five longest *B. xylophilus* scaffolds according to the *C. elegans* chromosome (WormBase WS221) on which their orthologue was found (Figure 3). This shows that, as for the comparison between *T. spiralis* and *C. elegans*, the *B. xylophilus* scaffolds show a macrosyntenic relationship with *C. elegans* chromosomes. In the case of *B. xylophilus* scaffold00713 for instance, many of the genes are orthologous to those from *C. elegans* chromosome V, but are interspersed with genes whose orthologues reside on all other *C. elegans* chromosomes.

1. Leroy S, Duperray C, Morand S (2003) Flow cytometry for parasite nematode genome size measurement. Mol Biochem Parasitol 128: 91-93.

2. Schattner P, Brooks AN, Lowe TM (2005) The tRNAscan-SE, snoscan and snoGPS web servers for the detection of tRNAs and snoRNAs. Nucleic Acids Res 33: W686-689.

3. Ellis RE, Sulston JE, Coulson AR (1986) The rDNA of C. elegans: sequence and structure. Nucleic Acids Res 14: 2345-2364.

4. Davey RP, James SA, Dicks J, Roberts IN (2010) TURNIP: Tracking Unresolved Nucleotide Polymorphisms in large hard-to-assemble regions of repetitive DNA sequence. Bioinformatics 26: 2908-2909.

5. Pettitt J, Harrison N, Stansfield I, Connolly B, M ller B (2010) The evolution of spliced leader trans-splicing in nematodes. Biochem Soc Trans 38: 1125-1130.

6. Mao X, Cai T, Olyarchuk JG, Wei L (2005) Automated genome annotation and pathway identification using the KEGG Orthology (KO) as a controlled vocabulary. Bioinformatics 21: 3787-3793.

7. van Megen H, van den Elsen S, Holterman M, Karssen G, Mooyman P, et al. (2009) A phylogenetic tree of nematodes based on about 1200 full-length small subunit ribosomal DNA sequences. Nematology 11: 927-950.

8. Mitreva M, Jasmer DP, Zarlenga DS, Wang Z, Abubucker S, et al. (2011) The draft genome of the parasitic nematode Trichinella spiralis. Nat Genet 43: 228-235.

9. Parkinson J, Mitreva M, Whitton C, Thomson M, Daub J, et al. (2004) A transcriptomic analysis of the phylum Nematoda. Nat Genet 36: 1259-1267.

10. Rawlings ND, Barrett AJ, Bateman A (2010) MEROPS: the peptidase database. Nucleic Acids Res 38: D227-233.

11. Jones JT, Kumar A, Pylypenko LA, Thirugnanasambandam A, Castelli L, et al. (2009) Identification and functional characterization of effectors in expressed sequence tags from various life cycle stages of the potato cyst nematode Globodera pallida. Mol Plant Pathol 10: 815-828.

12. Park SK, Tedesco PM, Johnson TE (2009) Oxidative stress and longevity in Caenorhabditis elegans as mediated by SKN-1. Aging Cell 8: 258-269.

13. Choe KP, Przybysz AJ, Strange K (2009) The WD40 repeat protein WDR-23 functions with the CUL4/DDB1 ubiquitin ligase to regulate nuclear abundance and activity of SKN-1 in Caenorhabditis elegans. Mol Cell Biol 29: 2704-2715.

14. Hasegawa K, Miwa J, Idnurm A (2010) Genetic and Cellular Characterization of Caenorhabditis elegans Mutants Abnormal in the Regulation of Many Phase II Enzymes. PLoS ONE 5: e11194.

15. Hasegawa K, Miwa J (2010) Transcriptome analysis of the xrep-1 (RNAi) phenocopy in C. elegans. Annual Report of Research Institute for Biological Function 10: 72-80.

16. An JH, Vranas K, Lucke M, Inoue H, Hisamoto N, et al. (2005) Regulation of the Caenorhabditis elegans oxidative stress defense protein SKN-1 by glycogen synthase kinase-3. Proc Natl Acad Sci U S A 102: 16275-16280.

17. Tullet J, Hertweck M, An JH, Baker J, Hwang JY, et al. (2008) Direct inhibition of the longevity-promoting factor SKN-1 by insulin-like signaling in C. elegans. Cell 132: 1025-1038.

18. Inoue H, Hisamoto N, An JH, Oliveira RP, Nishida E, et al. (2005) The C. elegans p38 MAPK pathway regulates nuclear localization of the transcription factor SKN-1 in oxidative stress response. Genes Dev 19: 2278-2283.

19. Hasegawa K, Miwa S, Isomura K, Tsutsumiuchi K, Taniguchi H, et al. (2008) Acrylamide-responsive genes in the nematode Caenorhabditis elegans. Toxicol Sci 101: 215-225.

20. Oliveira RP, Abate JP, Dilks K, Landis J, Ashraf J, et al. (2009) Condition-adapted stress and longevity gene regulation by Caenorhabditis elegans SKN-1/Nrf. Aging Cell 8: 524-541.

21. Dieterich C, Clifton SW, Schuster LN, Chinwalla A, Delehaunty K, et al. (2008) The Pristionchus pacificus genome provides a unique perspective on nematode lifestyle and parasitism. Nat Genet 40: 1193-1198.

22. Goldstein B, Macara IG (2007) The PAR proteins: fundamental players in animal cell polarization. Dev cell 13: 609-622.

23. Brauchle M, Kiontke K, MacMenamin P, Fitch DHA, Piano F (2009) Evolution of early embryogenesis in rhabditid nematodes. Dev Biol 335: 253-262.

24. Hu PJ (2007) Dauer. WormBook 25: 1-29.

25. Ogawa A, Streit A, Antebi A, Sommer RJ (2009) A conserved endocrine mechanism controls the formation of dauer and infective larvae in nematodes. Curr Biol 19: 67-71.

26. Viney ME (2009) How did parasitic worms evolve? BioEssays 31: 496-499.

27. Joo HJ, Yim YH, Jeong PY, Jin YX, Lee JE, et al. (2009) Caenorhabditis elegans utilizes dauer pheromone biosynthesis to dispose of toxic peroxisomal fatty acids for cellular homoeostasis. Biochem J 422: 61-71.

28. Joo HJ, Kim KY, Yim YH, Jin YX, Kim H, et al. (2010) Contribution of the Peroxisomal acox Gene to the Dynamic Balance of Daumone Production in Caenorhabditis elegans. J Biol Chem 285: 29319-29325.

29. Maehara N, Futai K (1996) Factors affecting both the numbers of the pinewood nematode, Bursaphelenchus xylophilus (Nematoda: Aphelenchoididae), carried by the Japanese pine sawyer, Monochamus alternatus (Coleoptera: Cerambycidae), and the nematode's life history. Appl Entomol Zoolog 31: 443-452.

30. Ishibashi N, Kondo E (1977) Occurrence and survival of the dispersal forms of pine wood nematode, Bursaphelenchus lignicolus Mamiya and Kiyohara. Appl Entomol Zoolog 12: 293-302.

31. Kikuchi T, Aikawa T, Kosaka H, Pritchard L, Ogura N, et al. (2007) Expressed sequence tag (EST) analysis of the pine wood nematode Bursaphelenchus xylophilus and B. mucronatus. Mol Biochem Parasitol 155: 9-17.

32. Mousley A, McVeigh P, Dalzell JJ, Maule AG (2011) Progress in the understanding of nematode neuropeptide communication systems. In: Kennedy MW, Harnett W, editors. Parasitic Nematodes, 2^nd^ Edition: Molecular Biology, Biochemistry and Immunology. Wallingford, UK: CABI.

33. Abad P, Gouzy J, Aury JM, Castagnone-Sereno P, Danchin EG, et al. (2008) Genome sequence of the metazoan plant-parasitic nematode Meloidogyne incognita. Nat Biotechnol 26: 909-915.

34. Bargmann CI (2006) Comparative chemosensation from receptors to ecology. Nature 444: 295-301.

35. Thomas JH, Robertson HM (2008) The Caenorhabditis chemoreceptor gene families. BMC biology 6: 42.

36. Benton R, Vannice KS, Gomez-Diaz C, Vosshall LB (2009) Variant ionotropic glutamate receptors as chemosensory receptors in Drosophila. Cell 136: 149-162.

37. Opperman CH, Bird DM, Williamson VM, Rokhsar DS, Burke M, et al. (2008) Sequence and genetic map of Meloidogyne hapla: A compact nematode genome for plant parasitism. Proc Natl Acad Sci U S A 105: 14802-14807.
