# Supplementary material for: Genomic Insights into the Origin of Parasitism in the Emerging Plant Pathogen Bursaphelenchus xylophilus
Source: PLoS Pathog. 2011 Sep 1;7(9):e1002219. doi: 10.1371/journal.ppat.1002219 (PMC3164644; doi:10.1371/journal.ppat.1002219)
Supplement: Text S2 — Supplementary tables. (DOC) [file ppat.1002219.s014.doc]

Table S1. Genome size estimation using Real-time PCR-based method^a^

| Species | Genomic DNA  concentration (ng/µl) | Target copies^b^ (copy number/µl)  (mean ± CE )^c^ | Estimated Genome size (bp/copy) |
| --- | --- | --- | --- |
| *B. xylophilus* (Ka4C1) | 134 | 1.80 * 10^6^ ±8% | 6.90 * 10^7^ ±8% |

a Welhen et. al [1], b Translation elongation factor 1 alpha (genbank no GU130155) was used as a reference. c CE (coefficient of error) = 100 * (standard error) * (mean)^-1^ (n = 3)

Table S2. Number of predicted tRNA genes

| UUU Phe | 0 |  |  | UCU Ser | 13 | (2) |  | UAU Tyr | 0 |  |  | UGU Cys | 0 |  |
| --- | --- | --- | --- | --- | --- | --- | --- | --- | --- | --- | --- | --- | --- | --- |
| UUC Phe | 13 | (1) |  | UCC Ser | 0 | (9) |  | UAC Tyr | 10 |  |  | UGC Cys | 8 | (1) |
| UUA Leu | 2 | (2) |  | UCA Ser | 3 | (1) |  | UAA end | 0 | (1) |  | UGA end | 0 |  |
| UUG Leu | 14 |  |  | UCG Ser | 4 | (1) |  | UAG end | 0 |  |  | UGG Trp | 8 |  |
| CUU Leu | 16 |  |  | CCU Pro | 8 |  |  | CAU His | 0 |  |  | CGU Arg | 10 |  |
| CUC Leu | 0 | (2) |  | CCC Pro | 0 | (27) |  | CAC His | 18 |  |  | CGC Arg | 0 |  |
| CUA Leu | 4 |  |  | CCA Pro | 23 | (1) |  | CAA Gln | 8 | (4) |  | CGA Arg | 7 |  |
| CUG Leu | 8 | (1) |  | CCG Pro | 3 |  |  | CAG Gln | 6 |  |  | CGG Arg | 0 |  |
| AUU Ile | 17 |  |  | ACU Thr | 12 | (2) |  | AAU Asn | 0 |  |  | AGU Ser | 0 |  |
| AUC Ile | 0 |  |  | ACC Thr | 0 | (1) |  | AAC Asn | 13 |  |  | AGC Ser | 8 |  |
| AUA Ile | 2 |  |  | ACA Thr | 5 | (1) |  | AAA Lys | 8 | (77) |  | AGA Arg | 4 | (1) |
| AUG Met | 14 |  |  | ACG Thr | 3 |  |  | AAG Lys | 14 |  |  | AGG Arg | 5 |  |
| GUU Val | 14 |  |  | GCU Ala | 16 | (2) |  | GAU Asp | 0 | (40) |  | GGU Gly | 0 | (111) |
| GUC Val | 0 | (1) |  | GCC Ala | 0 | (2) |  | GAC Asp | 16 | (1) |  | GGC Gly | 15 | (2) |
| GUA Val | 5 |  |  | GCA Ala | 4 | (1) |  | GAA Glu | 15 | (3) |  | GGA Gly | 15 | (1) |
| GUG Val | 5 |  |  | GCG Ala | 3 |  |  | GAG Glu | 14 | (1) |  | GGG Gly | 2 |  |

The numbers of tRNA pseudogenes are shown in parentheses.

Table S3. Statically significant (<0.05) differences in pathways in *B. xylophilus* compared to *C. elegans* identified by KOBAS

1. Pathways more prominent in *B. xylophilus* than *C. elegans*

| **KO Term** | **ID** | ***B. xylophilus*** | ***C. elegans*** | **P-value** | **Q-value** |
| --- | --- | --- | --- | --- | --- |
| Lysosome | ko04142 | 230 / 3392 | 125 / 3891 | 2.57E-12 | 6.58E-10 |
| Metabolism of xenobiotics by cytochrome P450 | ko00980 | 94 / 3392 | 52 / 3891 | 1.92E-05 | 2.46E-03 |
| Drug metabolism - cytochrome P450 | ko00982 | 92 / 3392 | 56 / 3891 | 1.72E-04 | 6.75E-03 |
| Fatty acid metabolism | ko00071 | 108 / 3392 | 87 / 3891 | 1.52E-02 | 1.69E-01 |
| Tyrosine metabolism | ko00350 | 58 / 3392 | 41 / 3891 | 2.08E-02 | 2.08E-01 |
| Glycosphingolipid biosynthesis - lacto and neolacto series | ko00601 | 19 / 3392 | 8 / 3891 | 2.20E-02 | 2.09E-01 |
| gamma-Hexachlorocyclohexane degradation | ko00361 | 53 / 3392 | 37 / 3891 | 2.44E-02 | 2.16E-01 |
| Phenylalanine metabolism | ko00360 | 50 / 3392 | 35 / 3891 | 3.02E-02 | 2.57E-01 |
| Glycosphingolipid biosynthesis - globo series | ko00603 | 9 / 3392 | 2 / 3891 | 4.11E-02 | 2.70E-01 |
| Tryptophan metabolism | ko00380 | 81 / 3392 | 66 / 3891 | 4.44E-02 | 2.73E-01 |
| Glycosaminoglycan degradation | ko00531 | 16 / 3392 | 7 / 3891 | 4.50E-02 | 2.73E-01 |
| ABC transporters | ko02010 | 35 / 3392 | 23 / 3891 | 4.79E-02 | 2.78E-01 |

1. Pathways less prominent in *B. xylophilus* than *C. elegans*

| **KO Term** | **ID** | ***B. xylophilus*** | ***C. elegans*** | **P-value** | **Q-value** |
| --- | --- | --- | --- | --- | --- |
| MAPK signaling pathway | ko04010 | 117 / 3392 | 209 / 3891 | 9.61E-05 | 6.15E-03 |
| Oocyte meiosis | ko04114 | 87 / 3392 | 157 / 3891 | 6.44E-04 | 1.94E-02 |
| Insulin signaling pathway | ko04910 | 88 / 3392 | 155 / 3891 | 1.25E-03 | 3.20E-02 |
| Melanogenesis | ko04916 | 73 / 3392 | 127 / 3891 | 4.74E-03 | 8.74E-02 |
| Long-term potentiation | ko04720 | 56 / 3392 | 103 / 3891 | 4.78E-03 | 8.74E-02 |
| Progesterone-mediated oocyte maturation | ko04914 | 58 / 3392 | 105 / 3891 | 5.68E-03 | 9.69E-02 |
| Olfactory transduction | ko04740 | 28 / 3392 | 59 / 3891 | 9.35E-03 | 1.33E-01 |
| ErbB signaling pathway | ko04012 | 56 / 3392 | 99 / 3891 | 1.07E-02 | 1.44E-01 |
| Calcium signaling pathway | ko04020 | 94 / 3392 | 150 / 3891 | 1.25E-02 | 1.60E-01 |
| Phosphatidylinositol signaling system | ko04070 | 49 / 3392 | 88 / 3891 | 1.34E-02 | 1.63E-01 |
| Wnt signaling pathway | ko04310 | 108 / 3392 | 165 / 3891 | 2.11E-02 | 2.08E-01 |
| mTOR signaling pathway | ko04150 | 32 / 3392 | 61 / 3891 | 2.37E-02 | 2.16E-01 |
| Cell cycle | ko04110 | 104 / 3392 | 157 / 3891 | 3.11E-02 | 2.57E-01 |
| Neurotrophin signaling pathway | ko04722 | 74 / 3392 | 117 / 3891 | 3.36E-02 | 2.59E-01 |
| Toll-like receptor signaling pathway | ko04620 | 26 / 3392 | 50 / 3891 | 3.97E-02 | 2.70E-01 |
| Naphthalene and anthracene degradation | ko00626 | 5 / 3392 | 17 / 3891 | 4.22E-02 | 2.70E-01 |
| Glycerophospholipid metabolism | ko00564 | 59 / 3392 | 95 / 3891 | 4.59E-02 | 2.73E-01 |

Table S4. Peptidases families detected by MEROPS bacth BLAST in *B. xylophilus* and other nematodes

|  | *B. xylophilus* | *M. incognita* | *M. hapla* | *C. elegans* | *P. pacificus* | *B. malayi* |
| --- | --- | --- | --- | --- | --- | --- |
| A01A | 67 (34) | 8 (4) | 5 (2) | 19 (15) | 32 (19) | 6 (3) |
| A11X | 1 (1) | 0 (0) | 0 (0) | 0 (0) | 0 (0) | 0 (0) |
| A22A | 3 (2) | 3 (2) | 4 (3) | 3 (3) | 2 (2) | 6 (6) |
| A22B | 2 (2) | 3 (3) | 2 (1) | 2 (2) | 1 (1) | 5 (5) |
| C01A | 65 (51) | 29 (20) | 20 (12) | 30 (26) | 24 (14) | 45 (15) |
| C02A | 6 (5) | 9 (6) | 5 (2) | 14 (10) | 5 (2) | 10 (7) |
| C12 | 3 (3) | 2 (1) | 2 (2) | 4 (4) | 2 (2) | 2 (2) |
| C13 | 15 (13) | 4 (2) | 1 (1) | 2 (2) | 2 (2) | 3 (3) |
| C14A | 1 (1) | 1 (1) | 1 (1) | 4 (3) | 1 (1) | 2 (2) |
| C14B | 1 (1) | 0 (0) | 1 (1) | 1 (1) | 1 (1) | 1 (1) |
| C15 | 1 (1) | 2 (2) | 0 (0) | 3 (3) | 0 (0) | 1 (1) |
| C19 | 20 (10) | 31 (11) | 12 (5) | 24 (15) | 18 (4) | 32 (13) |
| C26 | 2 (1) | 2 (2) | 1 (0) | 2 (1) | 2 (1) | 1 (1) |
| C44 | 8 (4) | 3 (1) | 1 (1) | 6 (2) | 4 (0) | 2 (1) |
| C46 | 3 (3) | 0 (0) | 1 (0) | 10 (9) | 10 (9) | 3 (2) |
| C48 | 5 (4) | 8 (4) | 2 (2) | 4 (4) | 4 (3) | 5 (5) |
| C50 | 1 (1) | 0 (0) | 1 (1) | 2 (2) | 0 (0) | 1 (1) |
| C54 | 2 (1) | 0 (0) | 1 (1) | 2 (2) | 1 (0) | 1 (1) |
| C56 | 2 (2) | 2 (2) | 1 (1) | 3 (3) | 1 (1) | 2 (2) |
| C64 | 1 (1) | 0 (0) | 0 (0) | 1 (1) | 1 (1) | 2 (1) |
| C65 | 1 (1) | 2 (2) | 1 (1) | 1 (1) | 1 (1) | 0 (0) |
| C67 | 0 (0) | 0 (0) | 0 (0) | 1 (1) | 0 (0) | 1 (1) |
| C74 | 0 (0) | 0 (0) | 0 (0) | 1 (0) | 0 (0) | 1 (0) |
| C78 | 1 (1) | 2 (2) | 1 (1) | 1 (1) | 1 (1) | 1 (1) |
| C83 | 0 (0) | 0 (0) | 0 (0) | 1 (1) | 1 (1) | 1 (1) |
| C85 | 1 (1) | 0 (0) | 0 (0) | 1 (0) | 0 (0) | 5 (3) |
| C86 | 2 (2) | 3 (3) | 2 (2) | 2 (2) | 2 (1) | 2 (2) |
| C89 | 1 (1) | 0 (0) | 1 (1) | 3 (3) | 7 (5) | 2 (0) |
| M01 | 26 (23) | 7 (6) | 5 (4) | 14 (13) | 13 (9) | 10 (6) |
| M02 | 1 (1) | 2 (2) | 1 (0) | 1 (1) | 1 (1) | 1 (1) |
| M03A | 3 (2) | 3 (2) | 2 (2) | 2 (2) | 2 (1) | 2 (2) |
| M08 | 2 (0) | 0 (0) | 1 (1) | 1 (1) | 1 (1) | 3 (2) |
| M10A | 6 (5) | 4 (4) | 0 (0) | 7 (7) | 6 (6) | 10 (10) |
| M12A | 25 (22) | 36 (34) | 19 (17) | 40 (40) | 52 (49) | 19 (17) |
| M12B | 9 (8) | 5 (5) | 4 (2) | 11 (11) | 12 (10) | 8 (8) |
| M13 | 52 (45) | 13 (11) | 8 (4) | 26 (21) | 21 (14) | 5 (2) |
| M14A | 10 (9) | 10 (6) | 6 (2) | 9 (9) | 9 (7) | 10 (3) |
| M14B | 4 (3) | 0 (0) | 2 (2) | 4 (4) | 4 (3) | 8 (8) |
| M14X | 1 (1) | 0 (0) | 0 (0) | 1 (1) | 0 (0) | 0 (0) |
| M16A | 0 (0) | 0 (0) | 0 (0) | 5 (5) | 1 (1) | 2 (1) |
| M16B | 4 (4) | 4 (4) | 3 (3) | 6 (6) | 8 (7) | 6 (5) |
| M16C | 2 (2) | 9 (7) | 1 (1) | 1 (1) | 1 (1) | 3 (2) |
| M17 | 2 (2) | 2 (2) | 1 (1) | 2 (2) | 5 (4) | 9 (2) |
| M18 | 1 (1) | 2 (1) | 2 (1) | 1 (1) | 1 (1) | 1 (1) |
| M20A | 7 (4) | 0 (0) | 3 (2) | 7 (7) | 5 (4) | 3 (3) |
| M22 | 3 (3) | 5 (5) | 3 (3) | 3 (3) | 2 (2) | 6 (6) |
| M23B | 0 (0) | 0 (0) | 0 (0) | 1 (1) | 1 (1) | 0 (0) |
| M24A | 2 (2) | 2 (2) | 1 (1) | 4 (2) | 2 (1) | 2 (2) |
| M24B | 4 (4) | 5 (5) | 2 (2) | 4 (4) | 3 (3) | 6 (4) |
| M24X | 2 (2) | 5 (5) | 1 (1) | 2 (2) | 3 (2) | 4 (3) |
| M28A | 1 (1) | 1 (1) | 1 (1) | 1 (1) | 0 (0) | 1 (1) |
| M28B | 3 (2) | 0 (0) | 2 (2) | 3 (3) | 4 (3) | 5 (1) |
| M28X | 2 (2) | 3 (3) | 1 (1) | 3 (3) | 3 (3) | 3 (3) |
| M38 | 4 (3) | 4 (2) | 5 (4) | 6 (6) | 5 (4) | 1 (1) |
| M41 | 4 (3) | 4 (4) | 5 (5) | 6 (5) | 2 (1) | 3 (3) |
| M43B | 0 (0) | 0 (0) | 0 (0) | 0 (0) | 0 (0) | 1 (1) |
| M48A | 1 (1) | 2 (2) | 2 (1) | 1 (1) | 2 (1) | 1 (1) |
| M49 | 1 (1) | 0 (0) | 0 (0) | 1 (1) | 5 (3) | 0 (0) |
| M50A | 1 (1) | 0 (0) | 1 (1) | 1 (1) | 1 (1) | 1 (1) |
| M67A | 5 (5) | 6 (1) | 5 (5) | 5 (5) | 7 (7) | 6 (6) |
| M67C | 0 (0) | 0 (0) | 0 (0) | 0 (0) | 0 (0) | 1 (1) |
| M67X | 2 (2) | 1 (1) | 2 (2) | 2 (1) | 2 (1) | 1 (1) |
| S01A | 3 (3) | 6 (3) | 4 (2) | 4 (4) | 18 (11) | 8 (5) |
| S01X | 1 (1) | 1 (1) | 0 (0) | 1 (1) | 1 (1) | 1 (0) |
| S08A | 4 (1) | 2 (0) | 0 (0) | 1 (1) | 2 (1) | 9 (2) |
| S08B | 3 (3) | 9 (4) | 2 (1) | 4 (4) | 3 (2) | 8 (5) |
| S09A | 1 (1) | 3 (2) | 0 (0) | 0 (0) | 2 (2) | 2 (2) |
| S09B | 2 (2) | 1 (0) | 2 (2) | 3 (3) | 4 (2) | 2 (2) |
| S09C | 5 (0) | 0 (0) | 3 (1) | 2 (2) | 3 (3) | 2 (1) |
| S09X | 62 (27) | 21 (10) | 24 (4) | 61 (39) | 65 (15) | 23 (12) |
| S10 | 23 (14) | 15 (5) | 4 (1) | 9 (9) | 17 (13) | 1 (1) |
| S12 | 6 (4) | 2 (1) | 0 (0) | 9 (8) | 8 (6) | 6 (4) |
| S14 | 1 (1) | 0 (0) | 1 (1) | 1 (1) | 1 (1) | 1 (1) |
| S16 | 2 (1) | 8 (8) | 2 (2) | 7 (7) | 1 (1) | 2 (1) |
| S26A | 2 (2) | 0 (0) | 1 (1) | 2 (2) | 1 (1) | 4 (2) |
| S26B | 1 (1) | 2 (2) | 1 (1) | 1 (1) | 2 (1) | 1 (0) |
| S28 | 16 (9) | 4 (3) | 2 (1) | 11 (9) | 16 (8) | 10 (1) |
| S33 | 18 (12) | 8 (4) | 4 (2) | 14 (13) | 14 (10) | 9 (6) |
| S54 | 3 (2) | 2 (1) | 2 (2) | 5 (5) | 7 (4) | 9 (5) |
| S59 | 1 (1) | 1 (1) | 1 (1) | 1 (1) | 1 (1) | 1 (1) |
| S63 | 1 (1) | 0 (0) | 0 (0) | 0 (0) | 0 (0) | 0 (0) |
| T01A | 14 (11) | 21 (13) | 14 (9) | 14 (13) | 12 (11) | 20 (14) |
| T02 | 1 (1) | 2 (2) | 2 (2) | 2 (2) | 1 (1) | 2 (2) |
| T03 | 5 (4) | 5 (3) | 31 (5) | 6 (4) | 7 (4) | 3 (3) |
| U48 | 1 (1) | 1 (1) | 1 (1) | 1 (1) | 1 (1) | 3 (3) |

Table S5. *C. elegans* homologous of dauer formation pathways in *B. xylophilus*. Yes indicates the presence of clear homologue gene; No indicates the absence.

| Guanylyl cyclase pathway |  |
| --- | --- |
| DAF-11 (Transmembrane guanylate cyclase) | Yes |
| TAX-2 (cGMP-gated channel) | Yes |
| TAX-4 (cGMP-gated channel) | Yes |
|  |  |
| TGFβ-like pathway |  |
| DAF-1 (TGFβ type I receptor) | Yes |
| DAF-3 (SMAD transcription factor) | Yes |
| DAF-4 (TGFβ type II receptor) | Yes |
| DAF-5 (Proline rich protein) | No |
| DAF-7 (TGFβ) | Yes |
| DAF-8 (SMAD transcription factor) | Yes |
| DAF-14 (SMAD transcription factor) | Yes |
| SCD-1 (Glutamine rich protein) | Yes |
| SCD-2 (Tyrosine kinase) | Yes |
| BRA-1 (Zn-finger protein) | Yes |
| KIN-8 (Tyrosine kinase) | Yes |
|  |  |
| Insulin/IGF pathway |  |
| DAF-2 (Insulin receptor) | Yes |
| DAF-18 (Phosphoinositide 3-phosphatase PTEN) | Yes |
| DAF-16 (FOXO transcription factor) | Yes |
| AGE-1 (Phosphoinositide 3-kinase) | Yes |
| PDK-1 (3-phosphoinositide-dependent kinase) | Yes |
| AKT-1 (Serine/threonine kinase) | Yes |
| AKT-2 (Serine/threonine kinase) | Yes |
| SGK-1 (Serine/threonine kinase) | Yes |
|  |  |
| Steroid holmone pathway |  |
| DAF-9 (Cytochrome P450) | Yes |
| DAF-12 (Nuclear receptor) | Yes |

Table S6. Neuropeptide-encoding *nlp* (neuropeptide-like protein), *ins* (insulin-like) and *flp* (FMRFamide-like peptide) genes in *B. xylophilus*. Grey shading indicates presence of orthologue of *C. elegans* gene; a,b denotes the existence of two *ins-1* subtypes; v denotes variant *ins-*like sequences (see methods and materials).

| **Gene number** | ***ins*** | ***Flp*** | ***Nlp*** |
| --- | --- | --- | --- |
| 1 | a,b |  |  |
| 2 |  |  |  |
| 3 |  |  |  |
| 4 |  |  |  |
| 5 |  |  |  |
| 6 |  |  |  |
| 7 |  |  |  |
| 8 |  |  |  |
| 9 |  |  |  |
| 10 |  |  |  |
| 11 |  |  |  |
| 12 |  |  |  |
| 13 |  |  |  |
| 14 |  |  |  |
| 15 |  |  |  |
| 16 |  |  |  |
| 17 | v |  |  |
| 18 |  |  |  |
| 19 |  |  |  |
| 20 |  |  |  |
| 21 |  |  |  |
| 22 |  |  |  |
| 23 |  |  |  |
| 24 |  |  |  |
| 25 |  |  |  |
| 26 |  |  |  |
| 27 |  |  |  |
| 28 |  |  |  |
| 29 |  |  |  |
| 30 |  |  |  |
| 31 |  |  |  |
| 32 | v |  |  |
| 33 |  |  |  |
| 34 |  |  |  |
| 35 |  |  |
| 36 |  |  |
| 37 |  |  |
| 38 |  |  |
| 39 |  |  |
| 40 |  |  |
| 41 |  |
| 42 |  |
| 43 |  |
| 44 |  |
| 45 |  |
| 46 |  |
| 47 |  |
|  | | | |

Table S7. Occurrence of orthologues of *C. elegans* biosynthesis and nuclear export of small RNA encoding genes in *B. xylophilus*. X indicates presence of putative orthologue.

| **Clade** | **Species** | **F26E4.10/*drsh-1*** | **T22A3.5/*pash-1*** | **K12H4.8/*dcr-1*** | **F15B10.2/*drh-1*** | **D2005.5/*drh-3*** | **T20G5.11/*rde-4*** | **ZK742.1/*xpo-1*** | **Y48G1A.5/*xpo-2*** | **C49H3.10/*xpo-3*** |
| --- | --- | --- | --- | --- | --- | --- | --- | --- | --- | --- |
| **IV** | ***Bursaphelenchus xylophilus*** | X | X | X | X | X | X | X | X |  |

Table S8. Occurrence of orthologues of *C. elegans* secondary amplification, uptake and intercellular spread of short interfering (si)RNA encoding genes in *B. xylophilus*. Shaded boxes indicate presence of orthologue; the number of predicted proteins that most-closely match the *C. elegans* search string is indicated.

.

|  |  | **Amplification components** | | | | | | | | **Spreading Components** | | |
| --- | --- | --- | --- | --- | --- | --- | --- | --- | --- | --- | --- | --- |
| **Clade** | **Species** | ***ego-1*** | ***smg-2*** | ***smg-5*** | ***smg-6*** | ***rrf-1*** | ***rrf-3*** | ***rsd-2*** | ***rsd-3*** | ***rsd-6*** | ***sid-1*** | ***sid-2*** |
| **IV** | ***Bursaphelenchus xylophilus*** | **4** | **1** |  | **1** | **2** | **3** |  |  | **1** |  |  |
|  | | | | | | | | | | | | |

Table S9. Occurrence of orthologues of *C. elegans* RNA-induced Silencing Complex (RISC) encoding genes in *B. xylophilus*. Shaded boxes indicate presence of orthologue; the number of predicted proteins that most-closely match the *C. elegans* search string is indicated. *C. elegans* pseudogenes not shown.

| **RISC Components** | | | | | | | | | | | | | | | | | | | | | | | | | | | | | | | | |
| --- | --- | --- | --- | --- | --- | --- | --- | --- | --- | --- | --- | --- | --- | --- | --- | --- | --- | --- | --- | --- | --- | --- | --- | --- | --- | --- | --- | --- | --- | --- | --- | --- |
| **Clade** | **Species** | **Argonautes** | | | | | | | | | | | | | | | | | | | | | | | | |  |  | **Non-Argonautes** | | | |
|  |  | **F48F7.1/alg-1** | **T07D3.7/alg-2** | **ZK757.3/tag-76/alg-4** | **F20D12.1/csr-1** | **R09A1.1/ergo-1** | **NRDE-3** | **C18E3.7/ppw-1** | **Y110A7A.18/ppw-2** | **D2030.6/prg-1** | **C01G5.2/prg-2** | **K08H10.7/rde-1** | **K12B6.1/sago-1** | **F56A6.1/sago-2** | **C04F12.1** | **C16C10.3** | **F55A12.1** | **F58G1.1** | **M03D4.7** | **R06C7.1** | **T22B3.2** | **T22H9.3** | **T23D8.7** | **Y49F6A.1** | **ZK1248.7** | **ZK218.8** |  |  | **F10G7.2/tsn-1** | **C06G1.4/ain-1** | **F56D12.5/vig-1** | **B0041.2/ain-2** |
| **IV** | ***Bursaphelenchus xylophilus*** | 1 | 1 |  | 2 |  |  |  |  |  |  | 1 |  |  |  |  |  |  |  | 6 | 1 |  | 1 | 1 | 2 |  |  |  | 1 | 1 | 1 |  |
|  | | | | | | | | | | | | | | | | | | | | | | | | | | | | | | | | |

Table S10. Occurrence of orthologues of *C. elegans* RNAi inhibitor encoding genes in *B. xylophilus*. Shaded boxes indicate presence of predicted orthologue; the number of predicted proteins that most-closely match the *C. elegans* search string is indicated.

| **Clade** | **Species** | **H15N14.1/*adr-1*** | **T20H4.4/*adr-2*** | **T07A9.5/*eri-1*** | ***eri-3*** | ***eri-5*** | ***eri-6/7*** | **ZK662.4/*lin-15b*** | **M04G12.4/*somi-1*** | **Y39G8C.1/*xrn-1*** | **Y48B6A.3/*xrn-2*** |
| --- | --- | --- | --- | --- | --- | --- | --- | --- | --- | --- | --- |
| **IV** | ***Bursaphelenchus xylophilus*** | 1 |  |  |  |  |  |  | 1 |  | 2 |

*Table S11. Occurrence of orthologues of C. elegans nuclear effector encoding genes in B. xylophilus. X indicates presence of predicted orthologue.*

| **Clade** | **Species** | ***cid-1*** | ***ekl-1*** | ***ekl-4*** | ***ekl-5*** | ***ekl-6*** | ***gfl-1*** | ***mes-2*** | ***mes-3*** | ***mes-6*** | ***mut-2*** | ***mut-7*** | ***mut-16*** | ***rde-2*** | ***rha-1*** | ***zfp-1*** |
| --- | --- | --- | --- | --- | --- | --- | --- | --- | --- | --- | --- | --- | --- | --- | --- | --- |
| **IV** | ***Bursaphelenchus xylophilus*** | **X** | **X** | **X** |  | **X** | **X** | **X** |  | **X** |  | **X** |  |  | **X** |  |
